# *Trichoderma*-Enabled Crop Resilience Under Abiotic Stress: From Field Delivery to Systems-Level Stress Reprogramming

**DOI:** 10.3390/jof12080578

**Published:** 2026-08-04

**Authors:** Xueping Su, Fangzhao Qin, Cheng Huang, Fasih Ullah Haider, Leiru Chen

**Affiliations:** 1 Hubei Key Laboratory of Biologic Resources Protection and Utilization, Hubei Minzu University, Enshi 445000, China; suxueping0504@mails.ccnu.edu.cn (X.S.); 13972441467@163.com (F.Q.); 2024102@hbmzu.edu.cn (C.H.); 2 South China Botanical Garden, Chinese Academy of Sciences, Guangzhou 510650, China; 3 Anhui Academy of Forestry, Hefei 230031, China

**Keywords:** *Trichoderma*, abiotic stress, crop resilience, salinity, drought, rhizosphere engineering

## Abstract

Abiotic stresses increasingly threaten crop productivity, whereas reliance on chemical and resource-intensive interventions can compromise environmental sustainability. Existing literature identifies *Trichoderma* spp. as multifunctional biocontrol agents, biofertilizers, and microbial biostimulants capable of influencing plant growth, stress signaling, and rhizosphere processes; however, evidence remains fragmented across strains, crops, formulations, and stress conditions. This review aimed to integrate current knowledge on *Trichoderma*-mediated resilience to salinity, drought, heavy metals, temperature extremes, and emerging pollutants, while distinguishing experimentally validated mechanisms from statistical associations and conceptual inference. It evaluates constraints governing reproducibility from controlled studies to field deployment. The synthesis shows that selected crop–strain systems improve root architecture, photosynthesis, antioxidant regulation, osmotic adjustment, nutrient acquisition, ion homeostasis, hormonal balance, and stress-responsive gene expression. Benefits arise through coordinated delivery, root colonization, metabolite and protein signaling, physiological reprogramming, and rhizosphere modulation. Nevertheless, microbiome co-occurrence patterns do not establish causal network repair, evidence for broad heat and cold protection remains limited, and biochar co-application should not be interpreted as a carrier formulation without direct validation. Future progress requires strain- and crop-specific screening, mechanistic gene and protein studies, standardized formulations, combined-stress experiments, multi-location field trials, biosafety evaluation, and farmer-level economic assessment to develop reliable precision microbial technologies.

## 1. Introduction

Abiotic stresses are major constraints to sustainable crop production and global food security [1,2]. Drought, salinity, heat, cold, flooding, nutrient imbalance, heavy-metal toxicity, and emerging pollutants such as microplastics disrupt plant growth across major crops, including rice (*Oryza sativa* L.), wheat (*Triticum aestivum* L.), maize (*Zea mays* L.), soybean (*Glycine max* L.), tomato (*Solanum lycopersicum* L.), common bean (*Phaseolus vulgaris* L.), and Indian mustard (*Brassica juncea* L.) [3,4,5]. These stresses impair seed germination, root development, photosynthesis, water relations, nutrient acquisition, and reproductive performance, ultimately reducing biomass and yield [6,7]. Drought limits water uptake, stomatal conductance and carbon assimilation; salinity causes osmotic stress and ion toxicity through excessive sodium (Na^+^) and chloride (Cl^−^) accumulation; temperature extremes disturb membrane stability and enzymatic processes; and heavy metals such as cadmium (Cd), lead (Pb), arsenic (As) and chromium (Cr) induce reactive oxygen species (ROS) accumulation, oxidative damage and metabolic dysfunction [8,9,10]. Because climate change is increasing the frequency and intensity of multiple stress events, crops are often exposed to combined stresses rather than single stress factors [3,11]. Therefore, mitigating abiotic stress while sustaining crop productivity is essential for resilient agriculture and long-term food security.

Several strategies have been used to improve crop tolerance to abiotic stresses, including conventional breeding, genetic engineering, irrigation and drainage management, mineral fertilization, organic amendments, biochar application, exogenous osmoprotectants, phytohormones, and physicochemical soil remediation [12,13,14,15,16]. Although these approaches have improved stress adaptation, they also face important limitations. Complex polygenic networks usually control stress tolerance and are strongly affected by genotype-by-environment interactions, making breeding slow, context-dependent, and difficult to translate across environments [12,13]. Chemical inputs and synthetic amendments can increase production costs and may contribute to soil degradation, secondary salinization, nutrient imbalance, and environmental pollution when poorly managed [14,15,16]. In contaminated soils, physicochemical remediation is often expensive and disruptive to soil structure, whereas phytoremediation alone can be limited by slow plant growth, insufficient biomass production, and low stress tolerance [10,17]. These limitations highlight the need for low-cost, eco-friendly, and biocompatible strategies that improve crop performance under stress while maintaining soil health.

Plant-associated microorganisms offer a promising route to strengthen crop resilience under adverse environments. Beneficial fungi and bacteria can enhance nutrient availability, stimulate root growth, regulate plant hormones, activate antioxidant defenses, improve water uptake, and restructure rhizosphere microbial communities [18,19,20]. Among them, *Trichoderma* spp. have received particular attention because of their multifunctional roles as biocontrol agents, biofertilizers, and microbial biostimulants [21,22,23]. Species such as *Trichoderma harzianum*, *Trichoderma asperellum*, *Trichoderma virens*, *Trichoderma atroviride*, *Trichoderma koningii*, and *Trichoderma longibrachiatum* can colonize the rhizosphere and root tissues of diverse crops, thereby creating a functional interface between the plant and soil microbiome [21,24]. Through this interface, *Trichoderma* can improve root architecture, nutrient uptake, chlorophyll retention, osmolyte accumulation, antioxidant enzyme activity, ion homeostasis, and stress-responsive gene expression [20,25]. For example, *T. harzianum* improved salt tolerance and yield in mustard by increasing chlorophyll, proline, antioxidant enzymes, and nutrient uptake, while reducing hydrogen peroxide (H_2_O_2_), malondialdehyde (MDA), Na^+^ accumulation, and the Na^+^/potassium (K^+^) ratio [25]. Similarly, *Trichoderma*-based treatments have improved drought tolerance, Cd stress resistance, and recovery from microplastic stress through physiological, biochemical, molecular, and rhizosphere-mediated mechanisms [5,17,26,27].

Despite rapid progress, the literature on *Trichoderma*-mediated abiotic stress tolerance remains fragmented. Many studies focus on a single crop, strain, or stress condition, whereas agricultural systems contain interacting stresses, diverse soils, and variable management practices [18]. Performance is strongly influenced by strain identity, host genotype, formulation or amendment type, inoculation method, soil physicochemical properties, indigenous microbial communities, and stress intensity [18,21,28,29,30]. The mechanisms connecting fungal metabolites, volatile compounds, secreted proteins, hormone-like signals, antioxidant pathways, aquaporins, salt overly sensitive signaling, heat-shock proteins, metal transporters, and treatment-associated microbiome patterns are not yet integrated into a sufficiently validated framework. This gap limits the translation of laboratory and pot studies into reliable field use. Accordingly, this review distinguishes experimentally measured plant responses from fungal-survival evidence, transgenic proof-of-concept, and statistical microbiome inference. It critically evaluates application strategies, root colonization, salinity and drought tolerance, heavy-metal stress and phytoremediation, temperature and emerging stresses, physiological and biochemical regulation, molecular and omics mechanisms, rhizosphere ecology, and microbial consortia. The resulting framework presents *Trichoderma* as a promising, strain- and context-dependent microbial technology whose broader crop protection and field reliability require direct validation.

## 2. Literature Search and Evidence-Synthesis Approach

This review adopted a structured narrative evidence-synthesis approach. Web of Science, Scopus, and PubMed were searched, with Google Scholar used to identify additional relevant studies, through 20 June 2026. Search strings combined “*Trichoderma*” with salinity, drought, heavy metal, temperature stress, pollutant, rhizosphere, microbiome, formulation, colonization, physiology, and omics using database-specific Boolean operators. No formal publication-year restriction was applied; recent studies were prioritized while relevant landmark studies were retained. The synthesis was based predominantly on English-language literature. Primary studies were prioritized for stress-specific, mechanistic, quantitative, and field evidence, whereas reviews were used for broader context. Studies focused exclusively on biotic control, lacking interpretable experimental detail, or duplicating previously reported datasets were excluded. Duplicate records were removed, overlapping reports were assessed jointly, and contradictory findings were interpreted according to strain identity, crop genotype, formulation or amendment type, inoculation method, soil properties, resident microbiota, and stress severity. Evidence was classified as field, greenhouse, pot, laboratory, fungal-genetic, purified-protein or peptide, transgenic or heterologous-expression proof-of-concept, amplicon-sequencing association, shotgun-metagenomic evidence, or conceptual inference.

## 3. The *Trichoderma* Platform: Genetic Capacity, Metabolic Signals, and Field Delivery

The agronomic value of *Trichoderma* spp. is best understood as a platform rather than as a single microbial input. Its capacity to reduce abiotic stress depends on the interactions among intrinsic strain traits, plant perception, rhizosphere establishment, and formulation design. Genome-era studies first expanded this view by showing that *Trichoderma* species are not only mycoparasites and enzyme producers, but also genetically versatile fungi with extensive secondary-metabolism pathways and numerous small secreted proteins that can participate in plant and microbial interactions [31]. More recent work on multicomponent inoculants, pan-genomics, and microbial consortia has further shifted attention from species names alone to strain-level functional capacity and ecological compatibility [28,32]. A coherent platform model must connect the genetic and metabolic capacity of *Trichoderma* with the delivery systems, carrier environments and ecological filters that determine field-level abiotic stress mitigation.

Stress resilience is unlikely to arise from a single protective pathway; rather, *Trichoderma*-mediated benefits depend on the sequential alignment of fungal traits, formulation performance, rhizosphere establishment and host perception. *Trichoderma*-mediated benefits emerge through a sequence of filters. A strain must contain relevant genetic and metabolic machinery. It needs to express useful metabolites, enzymes, or surface proteins in the target environment, survive storage and application, colonize the root zone, and interact productively with the resident microbiome. Failure at any stage can explain why responses observed in controlled pot experiments are sometimes weaker or inconsistent in field conditions. Thus, *Trichoderma* is best interpreted as a strain-specific, metabolically active and formulation-dependent biological platform whose field expression depends on whether genetic potential is preserved, delivered and established at the plant–soil interface.

### 3.1. Pan-Genome Architecture and Strain-Level Functional Capacity

The genus *Trichoderma* is taxonomically diverse, genetically plastic, and widely distributed across soil, rhizosphere, endosphere, and decomposing organic substrates. Current taxonomic concepts recognize more than 400 described species, and members of the genus occur across several major phylogenetic clades [28]. This diversity matters for abiotic stress research because the functional traits associated with root colonization, secondary metabolism, nutrient mobilization, and stress adaptation are not evenly distributed among all isolates. Therefore, statements such as “*Trichoderma* improves stress tolerance” should be interpreted with caution unless the strain, host crop, delivery system, and stress context are clearly defined.

Pan-genome analysis provides a powerful framework for explaining this strain-level heterogeneity. The core genome contains genes shared across strains and supports essential biological processes, whereas accessory and unique genes are more likely to encode niche-adaptive traits, including secondary metabolism, host interaction, microbial competition, and environmental responsiveness [32]. A recent pan-genome analysis of 25 agriculturally and industrially relevant *Trichoderma* strains identified 4960 shared core genes and revealed an open pan-genome, indicating continuing genetic innovation as additional strains are sampled [32]. The same study showed that industrial strains, such as *T. reesei* QM6a, possess unique features associated with cellulase secretion and lignocellulose degradation, whereas biocontrol-associated strains, such as *T. harzianum* CBS226.95 and *T. virens* Gv29-8, carry expanded accessory repertoires enriched in defense-related and secondary-metabolism functions [32].

For abiotic stress applications, this architecture has two implications. First, tolerance to salinity, drought, heavy metals, temperature extremes, or oxidative stress is unlikely to be predicted by a single marker. It probably arises from coordinated modules controlling osmotic adaptation, nutrient acquisition, detoxification, secreted proteins, signaling metabolites, and rhizosphere persistence. Second, strain selection should move beyond rapid growth or pathogen antagonism and include genomic indicators of stress fitness, root colonization, secondary-metabolite potential, transporter repertoires, and compatibility with formulation carriers. In this sense, pan-genomics provides an upstream selection logic for downstream bioformulation.

### 3.2. Secondary Metabolites as Plant-Regulating Signals

The metabolic capacity of *Trichoderma* is central to its biological activity. Genome-era studies and metabolite surveys show that *Trichoderma* species produce diverse biosynthetic gene clusters (BGCs), including non-ribosomal peptide synthetases (NRPSs), polyketide synthases (PKSs), terpene pathways, peptaibols, and other hybrid metabolite systems [32,33]. Many of these compounds were first studied as antifungal metabolites, but their relevance extends beyond antifungal activity: they can also influence plant development, stress priming, nutrient acquisition, and microbial communication [33,34]. This is why the secondary metabolome should be treated as part of the plant-stress regulatory interface, rather than solely as a biocontrol weapon.

Several *Trichoderma*-derived signals are directly relevant to abiotic stress tolerance. Indole-3-acetic acid (IAA) and related indolic compounds can stimulate root branching and increase the absorbing surface available for water and nutrient acquisition. Volatile organic compounds (VOCs), including 6-pentyl-2H-pyran-2-one (6-PP), sesquiterpenes, and ethylene-related signals, can promote root morphogenesis, chlorophyll accumulation, biomass formation, and defense-related gene expression [35]. In *Arabidopsis thaliana*, 6-PP from *T. atroviride* regulates root morphogenesis through auxin signaling and ETHYLENE INSENSITIVE 2 (*EIN2*) function, providing a mechanistic link between fungal volatiles and plant hormone pathways [36]. Other reviews report that *Trichoderma* can produce hormone-like metabolites, such as IAA, within biologically relevant ranges and release VOCs that can act without direct physical contact between the fungus and the plant [18].

The effects of these compounds are dose-, host-, and context-dependent. A metabolite that promotes root growth in one system may have weaker or different effects under another crop, soil, or stress regime. Therefore, future studies should quantify metabolite production under realistic rhizosphere conditions and link chemical profiles to plant outcomes, such as root hydraulic conductance, antioxidant capacity, ion balance, and yield stability. This would move the field from listing metabolites toward understanding which chemical signals matter under specific abiotic stresses.

### 3.3. Secreted Enzymes, Hydrophobins, and Nutrient-Mobilizing Traits

The *Trichoderma* secretome provides a second mechanistic layer connecting fungal activity with plant and rhizosphere function. Cell-wall-degrading enzymes such as chitinases, beta-1,3-glucanases, cellulases, xylanases, proteases, and other hydrolytic enzymes are classically associated with mycoparasitism and pathogen suppression [31,33]. In stress-prone agricultural soils, these enzymes may also contribute indirectly to abiotic stress tolerance by decomposing organic matter, releasing nutrients, modifying microbial competition, and supporting rhizosphere turnover. Small secreted cysteine-rich proteins and elicitor-like molecules can further influence plant immune priming and stress-responsive signaling [31].

Hydrophobins are particularly relevant because they connect fungal environmental fitness with root-associated performance. These small secreted cysteine-rich proteins modify fungal surface properties and support attachment, aerial growth, interface formation, and environmental persistence. HFB7, an orphan hydrophobin restricted to the Harzianum and Virens clades, is induced during interactions with plants, other fungi, and diverse abiotic stress conditions, suggesting a role in fungal stress adaptation and establishment [37]. Similarly, *TasHyd1* from *T. asperellum* is involved in plant root colonization, suggesting that hydrophobins can influence *Trichoderma*’s physical capacity to persist at the plant interface [38]. These traits are often overlooked in abiotic stress models, but they are important because plant benefits depend on stable fungal establishment under fluctuating soil moisture, salinity, temperature, and oxidative pressure.

Nutrient-mobilizing traits are also central to the *Trichoderma* platform. A classic study showed that *T. harzianum* Rifai 1295-22 can solubilize phosphates and micronutrients, linking fungal activity with improved nutrient availability [39]. This capacity is especially relevant under drought, salinity, and heavy-metal stress, where nutrient uptake is frequently constrained by poor root growth, altered rhizosphere chemistry, or ion antagonism. Together, secreted enzymes, hydrophobins, and nutrient-mobilizing functions explain why *Trichoderma* performance should be evaluated not only by plant biomass, but also by colonization stability, nutrient-use efficiency, and rhizosphere functional activity.

### 3.4. Formulation and Delivery as Determinants of Field Expression

Genetic and metabolic capacity cannot improve crop performance unless the inoculant is delivered effectively. *Trichoderma* can be applied through seed coating, seed biopriming, root dipping, soil drenching, liquid conidial suspensions, granular products, compost-based carriers, biochar-supported formulations, and multicomponent inoculants [28,29,30]. Each delivery method creates a different ecological entry point into the plant–soil system. Seed treatments favor early spermosphere and seedling-root colonization; root dipping is useful for transplanted crops; soil drenching places propagules directly into the rhizosphere; and carrier-based systems can protect fungal propagules while improving the local soil habitat.

Carrier design is therefore a biological determinant of performance rather than a simple technical detail. Talc, peat, alginate, clay minerals, compost, organic residues, and biochar differ in water retention, porosity, nutrient content, cost, shelf life, and compatibility with farm operations [29]. Bioformulation research emphasizes that shelf life and ecosystem adaptation must be considered together: an inoculant must remain viable during storage, activate after application, and retain function under local soil conditions [30]. This point is particularly important for abiotic stress mitigation because drought, salinity, and temperature extremes can directly reduce inoculum survival before plant benefits are expressed.

This formulation logic helps explain the frequent gap between controlled-environment studies and field reliability. Pot experiments often use simplified soils, regular irrigation, and high inoculum density; field soils, in contrast, contain diverse resident microbiota, variable moisture, fluctuating temperatures, and spatially heterogeneous nutrients. Stronger field translation will require carrier-only controls, live-inoculum treatments, viability measurements, colonization assays, and multi-location validation. Formulation should therefore be treated as part of the mechanism, because carrier quality, propagule viability and delivery route determine whether the genetic and metabolic potential of *Trichoderma* is expressed under field-relevant soil conditions.

### 3.5. Microbial Consortia and Compatibility with the Resident Microbiome

Co-inoculation with compatible microbial partners, including beneficial bacteria and fungi, can expand the functional breadth of *Trichoderma*-based technologies. Bacterial partners such as *Bacillus*, *Pseudomonas*, *Rhizobium*, *Bradyrhizobium*, and *Azospirillum* can contribute nitrogen fixation, phosphorus solubilization, siderophore production, phytohormone synthesis, biofilm formation, and pathogen suppression [40,41]. Such consortia may be especially useful under abiotic stress, such as drought, salinity, nutrient deficiency, and heavy metals, which simultaneously affect multiple plant functions. A compatible microbial consortium can therefore provide functional redundancy and complementary mechanisms that a single strain cannot. A drought study in *Astragalus mongholicus* further showed that dual inoculation with a dark septate endophyte and *T. viride* altered plant performance and rhizosphere microbiome responses [42].

However, microbial consortia should be designed rather than simply mixed. *Trichoderma*-bacterial interactions can be synergistic, neutral, or antagonistic depending on strain identity, nutrient competition, metabolite exchange, root niche overlap, and environmental conditions [40,41]. Compatibility must therefore be tested at three levels: microbial growth compatibility, formulation stability, and plant-response consistency. Recent reviews on multicomponent inoculants and *Trichoderma*-bacterial networks emphasize that stable consortia require attention to ecological interactions, carrier design, and predictable performance [28,40,41]. This is particularly relevant for abiotic stress applications, where environmental pressures can alter microbial behavior and shift the balance between cooperation and competition.

**Figure 1:**
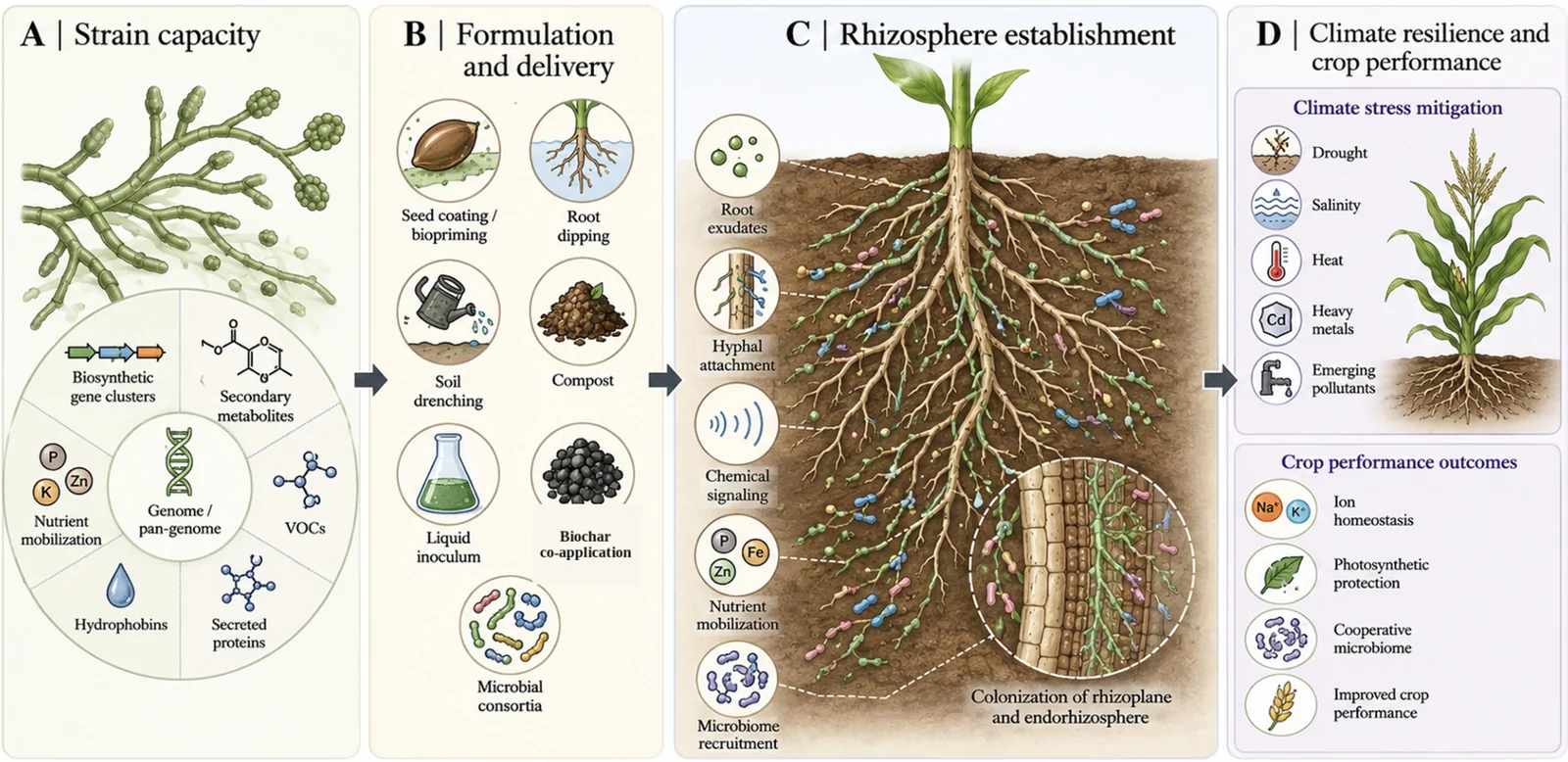
*Trichoderma* genetic capacity, delivery routes, and rhizosphere establishment as determinants of abiotic stress mitigation. The schematic presents a conceptual sequence from (**A**) strain-level functional capacity, including pan-genome diversity, biosynthetic gene clusters, secondary metabolites, hydrophobins, secreted proteins, volatile organic compounds, and nutrient-mobilizing traits; through (**B**) formulation and delivery approaches, including seed coating or biopriming, root dipping, soil drenching, compost incorporation, liquid inoculum, biochar co-application, and microbial consortia; to (**C**) rhizosphere establishment through root-exudate-mediated attraction, hyphal attachment, chemical signaling, nutrient mobilization, microbiome recruitment, and controlled colonization of the rhizoplane and endorhizosphere; ultimately contributing to (**D**) mitigation of drought, salinity, heat, heavy-metal, and emerging-pollutant stresses and to improved ion homeostasis, photosynthetic protection, cooperative microbiome interactions, and crop performance. The arrows indicate the conceptual progression among these functional stages. The symbols and icons represent the biological components and processes identified by their adjacent labels. Colors are used solely to distinguish panels and functional categories and do not indicate quantitative values, treatment intensity, or statistical differences. Original author-created schematic; not adapted from published material. Details of AI-assisted preliminary layout and author-led finalization are provided in the Declaration of Generative AI and AI-Assisted Technologies.

Overall, the *Trichoderma* platform should be understood as a series of interconnected biological filters rather than a single microbial input. Genomic and metabolic traits define the functional potential of a strain; formulation and delivery determine whether that potential survives storage, application, and entry into the root zone; root colonization and microbiome compatibility determine whether the strain becomes ecologically established; and plant perception determines whether stress-resilience pathways are activated. This strain-to-interface sequence is summarized in Figure 1, which integrates *Trichoderma* genetic capacity, metabolic signaling, formulation design, delivery routes, and root–rhizosphere establishment. Table 1 complements this model by linking major genomic, metabolic, secreted, nutritional, and formulation-related traits to their abiotic stress functions. This integrated view provides the foundation for the following sections, which examine how successful establishment at the plant–soil interface is translated into physiological, molecular, and rhizosphere-mediated crop resilience.

## 4. The *Trichoderma*-Plant Interface: Colonization, Signaling, and Rhizosphere Establishment

The transition from inoculation to abiotic stress mitigation depends on the formation of a functional interface at the root–soil boundary. This interface includes the spermosphere, rhizosphere, rhizoplane, and endorhizosphere, where plant exudates, fungal hyphae, and resident microorganisms interact in a chemically active microenvironment [18,21]. At this root–soil boundary, the functional potential of *Trichoderma* becomes biologically active only if conidia or hyphae perceive root-derived signals, attach to the rhizoplane, proliferate under local soil constraints and establish a balanced association that stimulates plant responses without imposing pathogenic costs. This distinction is central for field translation because inconsistent root establishment is one reason why strong pot-scale effects are not always reproduced under field conditions [28,29].

Root colonization begins with reciprocal chemical recognition. Plants release carbohydrates, amino acids, organic acids, lipids, and phenolic compounds into the rhizosphere, and these exudates act as nutrient sources, chemoattractants, and ecological filters for beneficial microorganisms [18]. Stress can further reshape this chemical landscape. For example, stressed plants may alter exudate profiles to increase the attraction or proliferation of beneficial fungi, including *Trichoderma* [18,21]. Once near the rhizoplane, *Trichoderma* hyphae attach to the root surface, grow along epidermal cells, and may form appressorium-like structures before entering superficial root tissues [24,43]. In classical interaction studies, *Trichoderma* colonization was associated with intercellular growth in the epidermis and outer cortex, while surrounding plant cells deposited wall material and phenolic compounds that restricted excessive fungal expansion [43]. This controlled accommodation explains why *Trichoderma* can behave as an opportunistic, avirulent symbiont rather than a pathogen [21,24]. Surface-active proteins are important in this colonization process. Hydrophobins modify fungal surface properties and can support attachment at hydrophobic-hydrophilic interfaces. The hydrophobin *TasHyd1* from *T. asperellum* is involved in plant root colonization, whereas HFB7, an orphan hydrophobin from the Harzianum and Virens clades, is induced during plant or fungal interactions and under abiotic stress [37,38]. These observations suggest that hydrophobins should be considered not only structural proteins but also ecological fitness factors that help *Trichoderma* persist at the root interface. This is relevant under drought, salinity, and fluctuating soil moisture, where stable attachment and hyphal persistence may determine whether downstream stress-mitigation traits are activated.

Chemical signaling provides the second layer of the interface. *Trichoderma* produces indole-3-acetic acid (IAA)-related indoles, 6-pentyl-2H-pyran-2-one (6-PP), volatile organic compounds (VOCs), peptaibols, siderophores, cell-wall-degrading enzymes, and small secreted proteins that can affect root development, defense priming, and stress physiology [33,34,35,36]. *Trichoderma*-derived indolic and volatile compounds can act at low concentrations and influence root development, defense priming, and hormone signaling [18,36,44,45,46,47]. Importantly, *Trichoderma*-mediated plant growth promotion can occur even without physical contact, showing that soluble and volatile signals can function as long-distance messengers in the soil air and soil solution [36,44].

The root developmental response is one of the clearest outcomes of this dialogue. Several *Trichoderma* strains stimulate lateral root formation, root hair proliferation, and primary root adjustment through auxin- and ET-related signaling [44,45,46,47]. In *Arabidopsis thaliana*, *Trichoderma virens* promoted biomass accumulation and lateral-root development through an auxin-dependent mechanism [45]. Similarly, the volatile 6-PP from *T. atroviride* regulates root morphogenesis through auxin signaling and ETHYLENE INSENSITIVE 2 functioning [36]. These responses are not only growth effects; they expand the nutrient- and water-absorbing surface that plants need under drought, salinity, and nutrient limitation. Thus, root architecture remodeling represents the structural bridge between fungal colonization and physiological stress tolerance.

Nutrient mobilization is the third component of the interface. *Trichoderma* can solubilize poorly available phosphate and micronutrients, secrete siderophores, produce organic acids, and contribute to organic matter decomposition through extracellular enzymes [18,31]. The classical study of *T. harzianum* Rifai 1295-22 demonstrated solubilization of phosphates and micronutrients, supporting a direct role for the fungus in nutrient acquisition [39]. This mechanism becomes especially important in stressed soils, where salinity, drought, pH imbalance, and heavy metals reduce nutrient availability or transport. Improved phosphorus (P), iron (Fe), zinc (Zn), manganese (Mn), and other nutrient fluxes can sustain chlorophyll formation, enzymatic activity, redox balance, and growth recovery. Therefore, nutrient mobilization should be viewed as part of abiotic stress mitigation, not merely as a general biofertilizer effect.

Rhizosphere establishment also changes the wider microbiome. *Trichoderma* can compete for space and nutrients, suppress pathogens, alter root exudation, and chemically interact with bacteria and fungi [48,49,50,51]. In the rhizosphere, it may recruit or cooperate with plant growth-promoting rhizobacteria, including *Bacillus*, *Pseudomonas*, *Rhizobium*, *Bradyrhizobium*, *Azospirillum*, and *Azotobacter* species [40,41,48]. Such interactions are not always positive; they range from synergistic to antagonistic depending on strain identity, metabolite production, and niche overlap [40,41]. This explains why microbial consortia require compatibility testing rather than simple mixing. For example, inoculation of *Trichoderma viride-Azotobacter chroococcum* biofilm has been reported to modulate rhizosphere colonization, plant growth, soil nutrient availability, and defense enzyme activity in chickpea [48]. These findings support the idea that the *Trichoderma*-plant interface is also a microbial-network interface.

Root-localized colonization can also generate systemic responses. Beneficial fungal colonization can reprogram plant gene expression and activate induced systemic resistance (ISR), systemic acquired resistance (SAR)-like pathways, antioxidant priming, and hormone crosstalk [21,43,52,53]. Although these mechanisms were originally studied mainly in disease resistance, their relevance extends to abiotic stress because many stress responses share regulatory modules, including ROS, calcium signaling, mitogen-activated protein kinase (MAPK) cascades, abscisic acid (ABA), jasmonic acid (JA), salicylic acid (SA), ET, and auxin pathways [18,21,54]. This helps explain why root-associated *Trichoderma* can influence photosynthesis, nutrient uptake, oxidative stress, and stress-responsive gene expression in distant tissues [18,20]. The interface, therefore, functions as a local perception site that can trigger whole-plant acclimation.

The final property of the interface is specificity. Colonization and signaling are shaped by fungal strain, crop genotype, plant age, inoculum density, soil texture, moisture, pH, organic matter, indigenous microbiota, and stress severity [18,21]. A strain selected for strong pathogen antagonism may not be optimal for drought or salinity mitigation, and a strain that performs well in sterile pot soil may fail in a field soil with high microbial competition or poor moisture. Likewise, a metabolite that promotes root branching at one concentration may inhibit growth at another. These context dependencies do not weaken the case for *Trichoderma*; rather, they define the conditions for precision deployment. Future studies should therefore report colonization intensity, persistence, root-zone localization, and microbiome response alongside plant growth and stress physiology.

The *Trichoderma*–plant interface is the critical checkpoint that determines whether delivered inoculum becomes an active stress-mitigation partner rather than a transient microbial input. A strain can carry useful genetic, metabolic, and stress-adaptive traits. Still, these traits become agronomically meaningful only when the fungus reaches the rhizosphere, attaches to the root surface, communicates through soluble and volatile signals, and establishes a balanced association with the plant and resident microbiota. Delivery, formulation, and root–rhizosphere establishment together form the first operational layer of *Trichoderma*-enabled crop resilience (Figure 1). Only when this interface is stable should downstream responses, including antioxidant defense, osmotic adjustment, ion homeostasis, metal detoxification, treatment-associated microbiome changes, and crop-performance outcomes, be attributed reproducibly to *Trichoderma* activity. The practical question is therefore not simply whether a strain possesses stress-mitigation traits, but whether those traits are expressed at the correct biological interface and remain active under the crop, soil, and stress conditions of the target production system.

## 5. Abiotic Stress Mitigation by *Trichoderma*

*Trichoderma*-mediated abiotic stress mitigation can be understood as a stress-specific response built on shared recovery modules that stabilize plant function across contrasting environmental constraints. Salinity, drought, heavy metals, temperature extremes, and emerging pollutants cause distinct primary injuries [1,3,4,5,10]. Still, they converge on disturbed water relations, ion and nutrient imbalances, impaired photosynthesis, membrane damage, oxidative stress, and rhizosphere disruption. *Trichoderma* does not remove the external stress; in experimentally supported crop systems, it can reduce the physiological cost of exposure by improving root function, nutrient mobilization, redox buffering, osmotic adjustment, hormonal balance, and rhizosphere processes [18,19,20,21]. The following subsections distinguish direct crop responses from fungal-survival evidence, transgenic proof-of-concept, and microbiome associations, with quantitative outcomes emphasized where available.

### 5.1. Salinity Stress

Salinity imposes two successive constraints. The first is osmotic: high external salt levels lower soil water potential and restrict water uptake. The second is ionic. Excessive sodium (Na^+^) and chloride (Cl^−^) disturb potassium (K^+^), calcium (Ca^2+^), and magnesium (Mg^2+^) nutrition, destabilize membranes, and inhibit photosynthesis. In salt-affected crops such as Indian mustard (*Brassica juncea* L.), common bean (*Phaseolus vulgaris* L.), citrus rootstocks, and model plants such as *Arabidopsis thaliana*, the visible outcomes include reduced germination, root growth, chlorophyll content, biomass, and yield [25,55,56,57,58,59]. Salinity also intensifies oxidative stress by increasing ROS, H_2_O_2_, MDA, and other markers of membrane damage [7,8]. The strongest evidence for *Trichoderma* under salinity points to three linked outputs: ion rebalancing, antioxidant protection, and recovery of photosynthetic activity. In naturally saline soil, *T. harzianum* applied as compost or suspension increased chlorophyll, proline, oil content, and nutrient accumulation in Indian mustard. It also reduced Na^+^ uptake and the Na^+^/K^+^ ratio. Compost-based application increased seed yield by about 23% relative to the untreated control. Oil content increased by 19–23.4% under salt stress [25]. In the Tori-7 genotype, the Na^+^/K^+^ ratio fell from 2.14 in the untreated control to about 0.92 under the strongest compost treatment. This illustrates how the fungal response led to ionic stabilization, not just growth stimulation [25]. Earlier work in Indian mustard also showed that *T. harzianum* mitigated NaCl stress by enhancing antioxidative defense and improving the uptake of essential elements [58].

Mechanistic evidence is now emerging from genetic and horticultural systems. In the salt-sensitive *A. thaliana* salt overly sensitive 1 (sos1) mutant, *T. harzianum* increased fresh weight, chlorophyll fluorescence, photosynthetic pigments, and transcripts related to ROS scavenging under 150 mM NaCl. It also enhanced proline, alanine, sucrose, and glucose accumulation, while restricting Na^+^ accumulation [55]. In citrus, *T. harzianum* increased plant height, stem diameter, leaf number, biomass, photosynthetic rate, stomatal conductance, intercellular carbon dioxide (CO_2_), transpiration, and chlorophyll content. It also increased nitrogen (N), phosphorus (P), Ca, Mg, zinc (Zn), and copper (Cu), lowered Na, and up-regulated salt overly sensitive (SOS), plasma membrane intrinsic protein (PIP), and tonoplast intrinsic protein (TIP) genes [56]. These data suggest that *Trichoderma* improves salinity tolerance by linking Na^+^ efflux, water transport, and nutrient acquisition. The value of *Trichoderma* becomes clearer when salinity overlaps with biotic stress. In common bean, combined salinity and *Sclerotinia sclerotiorum* reduced germination to 47.5–50.0% in two cultivars. *T. harzianum* increased germination under combined stress to 96.0–97.0%. Damping-off under combined stress decreased from 52.5% and 50.0% to 14.5% and 10.8% after *T. harzianum* treatment [57]. The same study showed that *Trichoderma* preserved chlorophyll, increased K^+^ uptake, restricted Na^+^ accumulation, and reduced H_2_O_2_, superoxide, hydroxyl radicals, MDA, and methylglyoxal [57]. Thus, salinity mitigation is not only an ionic effect. It is an integrated process involving ion homeostasis, osmotic adjustment, antioxidant defense, photosynthetic maintenance, and stress-disease buffering.

### 5.2. Drought and Water Deficit

Drought affects crop growth by lowering soil water availability, reducing root hydraulic conductance, restricting stomatal opening, and limiting carbon assimilation [6,14]. It also reduces nutrient diffusion to the root zone and disrupts internal nutrient transport. In major crops, including rice (*Oryza sativa* L.), maize (*Zea mays* L.), sugarcane (*Saccharum officinarum* L.), tomato (*Solanum lycopersicum* L.), and tobacco (*Nicotiana tabacum* L.), drought tolerance depends on the integration of root architecture, water-use efficiency, osmoprotectants, antioxidant defense, and abscisic acid (ABA)-mediated stomatal regulation [6,14,60,61,62]. *Trichoderma* can reduce drought injury by expanding the functional root system and improving water and nutrient acquisition. Seed or root inoculation with *T. asperellum* T34 protected maize against drought and maintained fungal populations through crop development [62]. *T. asperellum* inoculation also attenuated drought stress in sugarcane by improving crop nutrition, chlorophyll and carotenoid concentrations, photosynthetic rate, stomatal conductance, and water-use efficiency. It increased superoxide dismutase (SOD), peroxidase (POD), proline, and sugar partitioning [63]. In rice, *T. harzianum* biopriming describes molecular programming under drought and supports the idea that early microbial exposure can prepare seedlings for later water deficit [60]. Foliar application of an endophytic *Trichoderma* biostimulant also increased drought resilience in maize and sunflower [64].

Water-transport traits provide a mechanistic bridge between fungal biology and plant drought performance. Heterologous overexpression of an aquaglyceroporin gene from *T. harzianum* improved water-use efficiency and drought tolerance in *N. tabacum*, providing transgenic proof-of-concept rather than evidence from direct fungal inoculation [61]. In tomato, *T. brevicompactum* altered physiological traits, ABA levels, and drought-marker gene expression, while *T. harzianum* treatments have been associated with increased secondary metabolites and proline under water deficit [65,66]. A controlled climate-room pot study in pepper (*Capsicum annuum* L.) evaluated biochar as a separately incorporated soil amendment and *T. harzianum* as an independently applied suspension; it therefore tested co-application, not a biochar-based fungal carrier or formulation [67]. Under 50% irrigation, the combined treatment increased CAT, POD, and SOD activities by more than 40% and reduced H_2_O_2_ by approximately 25%. In contrast, MDA was substantially higher in the combined-treatment group than in the water-stressed control (approximately 49.4 versus 16.8 mg g^−1^ fresh weight in the reported dataset) [67]. This discordance indicates that enhanced enzymatic ROS control and lower H_2_O_2_ levels did not translate into lower lipid peroxidation markers. The original authors proposed increased metabolic activity and lipid turnover as a possible explanation, but that mechanism was not experimentally resolved. Because the experiment was short-term, pot-based, and conducted in one soil under controlled conditions, it does not establish carrier-mediated protection, field performance, or generalized drought resilience.

### 5.3. Heavy-Metal Stress and Phytoremediation

Heavy metals such as cadmium (Cd), lead (Pb), chromium (Cr), arsenic (As), copper (Cu), and nickel (Ni) disrupt nutrient homeostasis in plants. They can replace essential metals in enzymes, damage membranes, and accelerate ROS production. Cd is particularly problematic because it is toxic at low concentrations, can move through the xylem, and can accumulate in edible tissues. Phytoremediation offers an ecological route for metal removal, but is often constrained by slow plant growth, low biomass, weak root systems, and stress-induced metabolic collapse [10,17]. *Trichoderma*-assisted phytoremediation should be considered a combined tolerance-and-removal strategy. In Cd-stressed Indian mustard, *T. harzianum* plus polyaspartic acid increased net photosynthetic rate, intercellular CO_2_, stomatal conductance, and transpiration by 63.29%, 48.95%, 33.73%, and 66.90%, respectively. Root length increased by 34.97% with *T. harzianum*. The combined treatment increased root volume and total biomass by 30.65% and 42.38%. Total Cd accumulation increased by 79.11%, and leaf Cd content increased by up to 71.12% [17]. These figures show that phytoremediation efficiency depended on restoring plant growth and transport capacity, not simply increasing metal availability.

Detoxification under Cd stress was linked to subcellular partitioning and antioxidant regulation. Cd was mainly localized in the cell wall and vacuolar or soluble fractions, while antioxidant enzymes and glutathione were associated with Cd distribution. Under *T. harzianum* plus polyaspartic acid, glutathione increased by 23.62% in leaves and 32.12% in roots, and metabolomic profiling detected 3525 root metabolites [17]. A companion study reported that the combined treatment increased soil Cd removal efficiency from 21.71% to 38.27%, cation-exchange capacity, organic matter, and Cd bioavailability [27]. Importantly, that study used 16S rRNA and ITS amplicon sequencing, along with co-occurrence network analysis, rather than shotgun metagenomic sequencing. It reported treatment-associated changes in bacterial and fungal community structure, network descriptors, and high-connectivity or core-associated taxa, including *Trichoderma*, *Pseudomonas*, and *Arenimonas*, alongside up-regulation of ZIP-family and TC.HME transporters [27]. These network results identify statistical associations and topological patterns; they do not, without experimental validation, establish causal fungal-led network repair, keystone function, or microbiome stability. The phytoremediation evidence therefore supports improved plant performance and treatment-associated rhizosphere restructuring, while the ecological mechanisms remain inferential.

### 5.4. Temperature Stress

Temperature extremes impose constraints on both crop plants and microbial inoculants. Heat stress destabilizes cellular membranes, disrupts protein folding, impairs photosynthetic machinery, and intensifies oxidative damage, whereas cold stress decreases membrane fluidity and restricts enzymatic activity, root metabolism, and nutrient acquisition [1,9,20]. Compared with salinity, drought, and heavy-metal stress, however, direct crop-level evidence for *Trichoderma*-mediated protection against heat or cold remains limited. Ahmed et al. [68] evaluated heat-treated *T. harzianum* strains and reported enhanced fungal growth following recovery, 50–58% polymorphism in the recovered material, and heat-shock protein bands of approximately 120 and 131 kDa. These findings provide evidence of fungal survival and post-stress adaptation but do not demonstrate enhanced crop heat tolerance, yield protection, or field-level reliability. Heat conditioning may therefore be useful as a preliminary strategy for identifying candidate thermotolerant strains; nevertheless, strains selected through fungal screening must subsequently be evaluated in plant-based experiments under controlled and field-relevant temperature regimes. Such studies should determine whether fungal thermotolerance translates into sustained inoculum viability, effective root colonization, reduced crop injury, and improved reproductive or yield performance. Ahmed et al. [68] investigated fungal adaptation rather than crop-level temperature protection.

Temperature adaptation may also influence *Trichoderma*’s ability to establish and persist at the plant–soil interface. HFB7, an orphan hydrophobin restricted to the Harzianum and Virens clades, is induced during interactions with plants and other fungi and in response to several abiotic stresses, suggesting a potential role in fungal environmental fitness and interface stability [37]. However, its direct contribution to crop protection under temperature stress has not yet been established. Similarly, the limited literature on *Trichoderma*-mediated cold tolerance proposes that antioxidant regulation, maintenance of membrane integrity, and improved root function are plausible protective mechanisms [20]. These processes should be presented as mechanistic hypotheses requiring experimental validation rather than as broadly demonstrated effects across crops. Future investigations should integrate measurements of inoculant viability, stress-conditioned propagule recovery, root colonization, and persistence with direct assessments of crop membrane injury, photosynthetic performance, antioxidant responses, recovery following stress removal, reproductive development, and yield. Experiments conducted under fluctuating temperature regimes and across multiple soils, crop genotypes, seasons, and field locations will be essential to determine whether fungal temperature adaptation can be translated into reproducible agronomic protection. This distinction is consistent with the manuscript’s conclusion that fungal survival following temperature exposure must not be treated as equivalent to crop protection.

### 5.5. Emerging, Nutrient-Related, and Combined Stresses

Agricultural soils are increasingly affected by stressors that do not fit neatly into traditional categories [3,5,11]. Microplastics, pesticide residues, acidic or alkaline soils, nutrient deficiency, and combined abiotic-biotic stress can disrupt root function, soil microbial balance, nutrient cycling, and redox homeostasis [3,5,8]. These stressors are important because they rarely act alone; they often interact with salinity, drought, heat, or pathogen pressure. *Trichoderma* is well-suited to such environments because its effects are distributed across plant metabolism, root architecture, microbial interactions, and soil biochemical activity.

Microplastic stress provides a recent example. Aged polybutylene adipate terephthalate (PBAT) microplastics inhibited *Nicotiana benthamiana* growth, increased ROS and MDA, and disrupted metabolic homeostasis. *T. harzianum* T4 reduced ROS/MDA, up-regulated SOD and POD activity, promoted biomass, reversed stress-disrupted gene-expression patterns, and reshaped the soil microbiome [5]. Metagenomic analysis showed increased relative abundances of *Bacteroidota* and *Myxococcota*, decreased relative abundances of *tetA5* and multidrug-resistance-related genes, and increased relative abundances of carbohydrate-active enzyme genes associated with carbon metabolism [5]. This expands *Trichoderma* research from conventional crop stress mitigation to ecological remediation of emerging pollutants.

**Figure 2:**
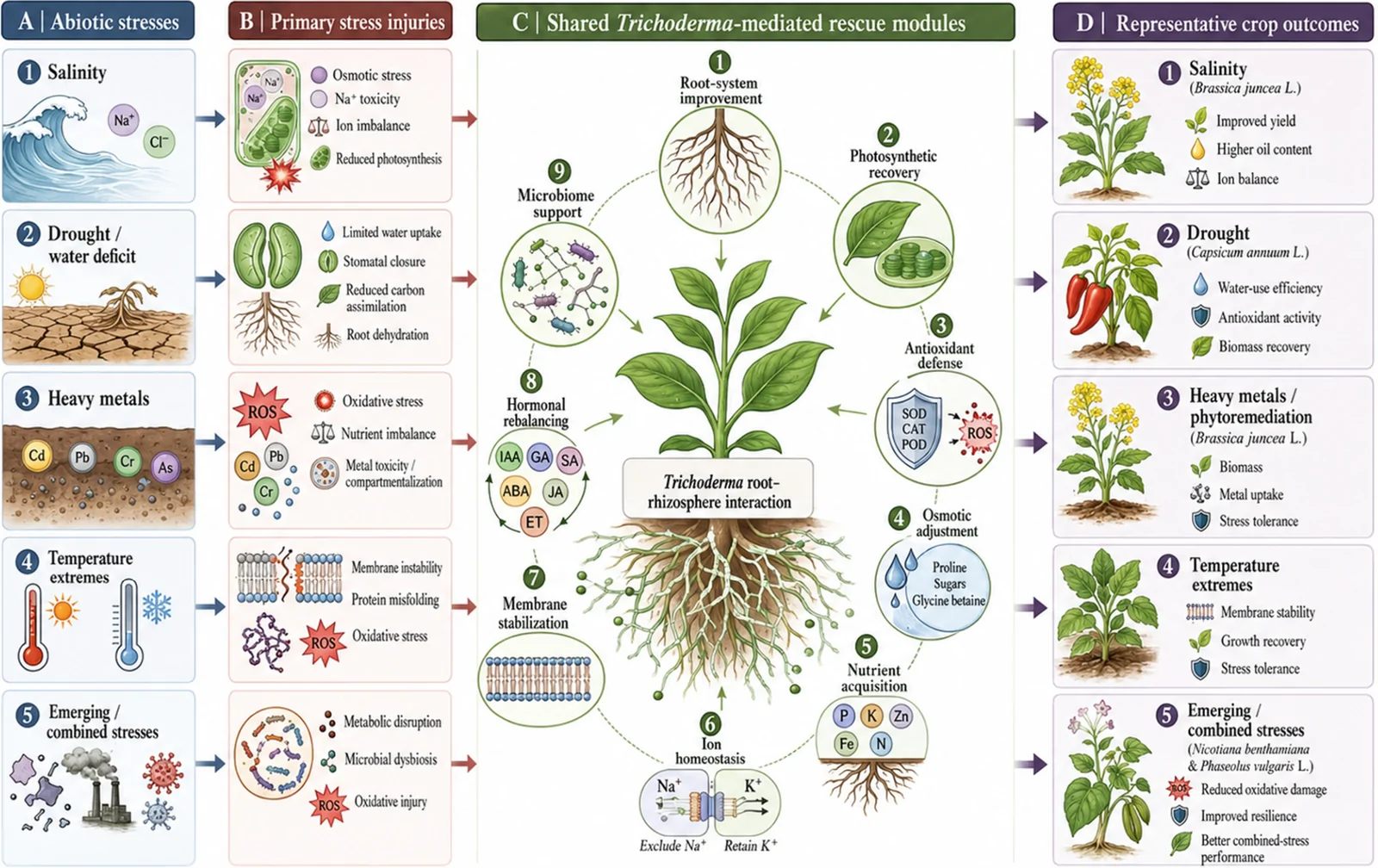
Stress-specific injuries and shared physiological rescue modules under *Trichoderma* treatment. **Panel A** presents five major abiotic-stress categories: salinity, drought or water deficit, heavy metals, temperature extremes, and emerging or combined stresses. **Panel B** illustrates their representative primary injuries, including osmotic and ionic stress, restricted water uptake and carbon assimilation, oxidative damage, nutrient imbalance, membrane instability, protein misfolding, metabolic disruption, and microbial dysbiosis. **Panel C** summarizes shared *Trichoderma*-mediated rescue modules, including root-system improvement, photosynthetic recovery, antioxidant defense, osmotic adjustment, nutrient acquisition, ion homeostasis, membrane stabilization, hormonal rebalancing, and microbiome support. **Panel D** presents representative reported or proposed crop outcomes under the corresponding stress categories. The salinity, drought, heavy-metal, and emerging-stress panels summarize representative outcomes reported in specific crop systems. The temperature-stress panel presents proposed plant-level mechanisms because direct crop evidence for broad heat- or cold-stress protection remains limited; fungal survival following temperature exposure should not be interpreted as evidence of crop protection. The blue, red, green, and purple panel backgrounds distinguish abiotic stresses, primary injuries, rescue modules, and crop outcomes, respectively. Numbered symbols link the corresponding stress categories and physiological modules, while the biological and chemical icons are identified by their adjacent labels. Arrows indicate the conceptual progression from stress exposure to injury and from *Trichoderma*-associated rescue processes to crop-level outcomes; they do not denote quantitative effects, treatment intensity, statistical significance, or universally established causal relationships. Original author-created schematic; not adapted from published material. Details of AI-assisted preliminary layout and author-led finalization are provided in the Declaration of Generative AI and AI-Assisted Technologies.

Combined stress remains an important frontier for *Trichoderma* research. The common-bean salinity-pathogen model shows that *Trichoderma* can reduce ionic stress, oxidative damage, and disease severity in a defined crop system [57]. Pepper and spinach studies evaluated the co-application of biochar as a soil amendment with independently applied *T. harzianum* [67,69]; these studies do not establish biochar as a fungal carrier. Heavy-metal studies link biomass recovery, transporter activity, subcellular compartmentalization, and treatment-associated microbiome changes [17,27], but co-occurrence-network metrics should be interpreted as statistical associations rather than proof of ecological repair or causality. Table 2 summarizes the stress-specific evidence and its experimental boundaries, whereas Figure 2 distinguishes directly measured crop outcomes from proposed temperature-related mechanisms that remain to be validated.

## 6. Physiological and Biochemical Reprogramming of Stressed Crops

Stress-specific responses described above converge on a smaller set of physiological and biochemical processes that determine whether stress injury becomes a recoverable state or a yield-limiting failure. *Trichoderma* spp. act at this level by shifting crops from damage accumulation toward regulated stress tolerance: photosynthetic apparatus is protected, redox metabolism is buffered, osmolytes accumulate, nutrient and ion balance is restored, membrane injury is reduced, and hormone networks are recalibrated. These physiological and biochemical processes provide the mechanistic bridge between stress exposure and measurable crop performance across salinity, drought, heavy-metal toxicity, temperature extremes and emerging pollutants. The emphasis is on markers that can be quantified across studies and used as translational indicators of *Trichoderma*-mediated stress resilience.

### 6.1. Photosynthetic Recovery and Carbon Assimilation

Photosynthesis is one of the earliest and most sensitive targets of abiotic stress. Drought restricts stomatal conductance and carbon dioxide (CO_2_) diffusion. Salinity reduces water potential and accelerates the development of sodium (Na^+^) toxicity. Heavy metals disturb chloroplast ultrastructure and electron transport. Temperature extremes destabilize photosystem function. These injuries result in lower chlorophyll content, reduced chlorophyll fluorescence, impaired gas exchange, and reduced biomass gain. *Trichoderma* inoculation can partially reverse this decline. It preserves pigments, improves root water acquisition, activates antioxidant protection around chloroplasts, and maintains carbon assimilation. A broad synthesis proposed that endophytic *Trichoderma* strains increase photosynthetic capability by changing chlorophyll, photosynthesis-related genes, and ROS-detoxifying pathways [70]. The quantitative evidence is increasingly crop-specific. In Indian mustard (*Brassica juncea* L.) under salinity, *Trichoderma harzianum* increased chlorophyll and oil content and improved seed yield. Compost-based application increased seed yield by 23% relative to the uninoculated control [25]. In cadmium (Cd)-stressed *B. juncea*, combined *T. harzianum* and polyaspartic acid treatment increased total chlorophyll by 37.10–91.98% and carotenoids by 34.78–72.37%. Total biomass increased by 42.38% [17]. In pepper (*Capsicum annuum* L.) exposed to water deficit, plants treated with combined biochar and *T. harzianum* were separated from stressed controls in principal component analysis based on growth promotion, antioxidant defense, and osmotic adjustment [67]. These findings indicate that photosynthetic recovery is not an isolated pigment response. It reflects coordinated protection of chloroplasts, water relations, nutrient status, and redox homeostasis.

### 6.2. Antioxidant Defense and Redox Buffering

Most abiotic stresses converge on oxidative stress. Excess ROS, including H_2_O_2_, superoxide, and hydroxyl radicals, damages proteins, nucleic acids, lipids, and membranes. *Trichoderma*-mediated redox responses commonly involve SOD, CAT, POD, APX, and glutathione reductase together with glutathione, ascorbate, phenolics, and flavonoids [7,26]. In salt-stressed Indian mustard, *T. harzianum* increased antioxidant enzyme activities and decreased H_2_O_2_ and MDA [25]. In Cd-stressed *B. juncea*, *T. harzianum* plus polyaspartic acid increased leaf CAT, SOD, and POD by 158.89%, 50.82%, and 6.71%, respectively [17]. The pepper co-application study produced a non-uniform redox response: CAT, POD, and SOD increased by more than 40%, and H_2_O_2_ decreased by approximately 25% under 50% irrigation, whereas MDA rose substantially relative to the water-stressed control [67]. Thus, this study supports improved enzymatic regulation of ROS but not reduced lipid peroxidation. Such contradictory markers should be discussed individually rather than combined into a generalized claim of complete oxidative-damage protection.

### 6.3. Osmotic Adjustment and Compatible Solutes

Osmotic adjustment is essential under drought and salinity. Compatible solutes such as proline, soluble sugars, alanine, amino acids, and soluble proteins stabilize macromolecules, maintain hydration, and contribute to ROS detoxification [62,71]. In salt-stressed *A. thaliana* sos1 mutants, *T. harzianum* increased proline, alanine, sucrose, and glucose while restricting Na^+^ accumulation [55]. In saline Indian mustard, *T. harzianum* also increased proline together with chlorophyll and antioxidant enzymes [25]. In pepper under severe water deficit, co-application of biochar and *T. harzianum* increased proline and sucrose [67]. Because biochar and the fungal suspension were applied independently, this result supports an amendment-microbe co-application effect and should not be described as carrier-fungus synergy.

### 6.4. Nutrient Uptake and Ion Homeostasis

Abiotic stress often disrupts nutrient acquisition. Salinity impairs potassium (K^+^) and calcium (Ca^2+^) uptake, leading to excessive accumulation of Na^+^ and chloride (Cl^−^). Drought limits nutrient diffusion to roots. Heavy metals interfere with essential transporters, and changes in pH or contaminant stress alter nutrient solubility. *Trichoderma* counters these effects through root architecture remodeling, organic acid production, phosphate solubilization, micronutrient mobilization, and regulation of ion transport [18,39]. The nutrient response is visible in crop studies. In saline Indian mustard, *T. harzianum* improved uptake and assimilation of nitrogen (N), phosphorus (P), sulfur (S), Ca, magnesium (Mg), and K. It also reduced Na uptake and lowered the Na^+^/K^+^ ratio [25]. In citrus seedlings, *T. harzianum* improved photosynthetic parameters, increased N, P, Ca, Mg, zinc (Zn), and copper (Cu), and reduced Na accumulation under salt stress [56]. Under Cd stress, the response is more complex. In *B. juncea*, combined *T. harzianum* and polyaspartic acid increased K, Na, and Mg accumulation by 42.28%, 63.58%, and 48.59%, respectively. Leaf Cd accumulation increased by 71.12% [17]. This apparent co-enrichment indicates that nutrient homeostasis during phytoremediation is not simply about metal exclusion. In hyperaccumulating or metal-remediating systems, *Trichoderma* may promote a coordinated state of nutrient-metal transport. This enables stress tolerance, compartmentalization, and biomass production, allowing greater contaminant removal without proportional growth inhibition.

### 6.5. Membrane Stability and Injury Limitation

Membrane stability integrates several injury processes. ROS attack polyunsaturated fatty acids, increasing MDA, electrolyte leakage, and loss of membrane selectivity. In several *Trichoderma*-treated crop systems, lower H_2_O_2_ and MDA have accompanied stronger antioxidant or osmolyte pools [25,26]. Kipçak Bitik et al. [67], however, reported lower H_2_O_2_ but substantially higher MDA after biochar-*T. harzianum* co-application under severe water deficit and therefore cannot be cited as evidence of reduced lipid peroxidation. In Cd-stressed *B. juncea*, *T. harzianum* plus polyaspartic acid shifted Cd toward cell-wall and vacuolar or soluble fractions, reducing exposure of sensitive organelles [17]. These results demonstrate that membrane-related outcomes are stress- and treatment-specific and should be assessed with multiple markers rather than inferred solely from antioxidant-enzyme activity.

### 6.6. Hormonal Regulation and Stress-Growth Trade-Offs

Stress tolerance requires rebalancing growth and defense through IAA, GA, ABA, SA, JA, and ethylene pathways [18,20,72]. In water-stressed pepper, the separate co-application of biochar and *T. harzianum* increased IAA and GA by more than 1500% and 2900%, respectively, decreased ABA by 99.5%, and increased SA and JA by up to 50% [67]. These values derive from a controlled pot experiment and indicate strong treatment-associated hormonal shifts, but they do not demonstrate a carrier-based fungal formulation or field-level recovery. Isolate-specific hormone profiles in melon further show that hormonal outcomes depend on the fungal strain, crop, and experimental context [72].

Collectively, these physiological and biochemical processes explain how root-associated *Trichoderma* activity is translated into measurable improvements in plant function and crop performance. Photosynthetic protection sustains carbon gain; antioxidant and osmolyte systems prevent irreversible cellular damage; nutrient and ion homeostasis supports metabolism; membrane stabilization maintains cellular integrity; and hormone rebalancing coordinates growth–defense trade-offs under stress. Figure 2 synthesizes this integrated physiological rescue model by showing how different abiotic stresses converge on shared recovery modules, including photosynthetic recovery, redox buffering, osmotic adjustment, nutrient acquisition, ion balance, membrane stability, and hormonal regulation. Table 3 provides practical markers for comparing *Trichoderma*-mediated stress responses across crops, stress types, and experimental systems.

## 7. Molecular and Omics Mechanisms

Physiological measurements show whether *Trichoderma*-treated plants recover under stress, but omics approaches explain how this recovery is coordinated across genes, metabolites, proteins, and microbial communities. The molecular evidence now indicates that *Trichoderma* does not simply increase a single defensive enzyme or metabolite; it reprograms interconnected regulatory modules that control ion transport, water movement, redox balance, hormone signaling, metal detoxification, and rhizosphere function. This systems perspective is essential because abiotic stress tolerance is not a single trait, but a network property emerging from the host plant, fungal strain, formulation context, and resident microbiome.

### 7.1. Stress-Responsive Gene Networks: From Markers to Modules

The most consistent molecular signature of *Trichoderma*-mediated stress mitigation is the activation or rebalancing of stress-responsive gene networks. Under salinity, genes involved in sodium exclusion, water transport, and antioxidant defense are particularly important. In citrus, *Trichoderma harzianum* inoculation under salt stress significantly upregulated salt overly sensitive (SOS) genes, plasma membrane intrinsic protein (PIP) aquaporins, and tonoplast intrinsic protein (TIP) genes, including TIP1, TIP4, and TIP9; these changes were associated with improved photosynthetic efficiency, nutrient uptake, sodium efflux, and water utilization [56]. This example links a clear molecular module with measurable physiological recovery: ion homeostasis, water transport, and carbon assimilation are regulated together rather than as isolated responses.

Drought responses show a similar network logic. Bioprimed rice (*Oryza sativa* L.) challenged by drought exhibited molecular programming associated with stress adaptation after *Trichoderma harzianum* treatment [60]. In tomato (*Solanum lycopersicum* L.), *Trichoderma brevicompactum* altered abscisic acid (ABA)-related responses and marker gene expression under drought, illustrating how fungal inoculation can influence both root and leaf signaling [66]. The importance of water-channel regulation is further supported by transgenic evidence: overexpression of a *Trichoderma harzianum* aquaglyceroporin improved water-use efficiency and drought tolerance in tobacco (*Nicotiana tabacum* L.) [61]. Together, these studies support a broader principle: *Trichoderma*-mediated drought tolerance involves root architecture and osmotic adjustment, but it is also encoded at the molecular level through aquaporin-linked water transport and hormone-responsive gene networks.

For heavy-metal stress, molecular regulation is more closely associated with detoxification, transport, and compartmentalization. Zinc-regulated transporter/iron-regulated transporter-like protein (ZIP) transporters and heavy-metal ATPases (HMAs) regulate metal entry, movement, and sequestration. In cadmium (Cd)-stressed Indian mustard (*Brassica juncea* L.), *Trichoderma harzianum* combined with polyaspartic acid (PASP) increased Cd tolerance and accumulation by coordinating antioxidant defense, nutrient uptake, subcellular Cd compartmentalization, and nutrient-Cd co-transport [17]. Metal tolerance should therefore be interpreted as a trade-off between phytoremediation uptake and detoxification via cell-wall binding, vacuolar sequestration, and glutathione-mediated chelation.

### 7.2. Transcriptomic Reprogramming Under Abiotic and Emerging Stresses

Transcriptomics has shifted the field from candidate-gene descriptions to pathway-level interpretation. In *Trichoderma*-treated plants, transcriptional changes typically involve transcription factors, antioxidant genes, aquaporins, transporters, defense-related genes, and hormone-responsive regulators. WRKY transcription factors, mitogen-activated protein kinase (MAPK) signaling components, pathogenesis-related (PR) proteins, and antioxidant genes such as superoxide dismutase (SOD), catalase (CAT), peroxidase (POD), ascorbate peroxidase (APX), and glutathione reductase (GR) create a regulatory bridge between stress perception and physiological protection [18,21,31]. Heat-shock proteins (HSPs) are also important stress-responsive components because they protect protein folding and cellular homeostasis during temperature and oxidative stress.

Emerging pollutants provide a useful test of this systems view. In *Nicotiana benthamiana* exposed to aged microplastics, *Trichoderma harzianum* T4 reversed gene-expression patterns disrupted by microplastic stress, particularly in pathways associated with deoxyribonucleic acid (DNA) replication and pentose-glucuronic acid metabolism [5]. These transcriptomic shifts coincided with lower ROS and MDA accumulation, improved antioxidant enzyme activity, and better plant biomass. The significance of this study lies not only in *Trichoderma* reducing microplastic injury, but also in transcriptomics revealing a coordinated recovery of growth, carbon metabolism, and stress-response pathways. A remaining challenge is that transcriptomic studies often use different crops, stress intensities, sampling times, and sequencing pipelines. This limits direct comparison among salinity, drought, heavy-metal, and pollutant studies. Future work should use time-resolved designs that separate early perception events from later acclimation responses. Simultaneously sampling roots, shoots, and rhizosphere tissues would also clarify whether *Trichoderma* first acts as a local root signal, a systemic plant regulator, or both.

### 7.3. Metabolomics: Chemical Evidence for Stress Reprogramming

Metabolomics provides the closest molecular link between gene regulation and plant performance [17,20]. Abiotic stresses frequently alter the levels of sugars, amino acids, organic acids, phenolics, flavonoids, glutathione, and other compatible solutes. These metabolites are not simply stress markers; they influence osmotic adjustment, redox buffering, membrane stability, metal chelation, and carbon allocation. In Cd-stressed *Brassica juncea* L., broad-spectrum metabolomic analysis detected 3525 metabolites across treatments; principal component analysis showed clear metabolic separation under the combined *Trichoderma harzianum*-PASP treatment, with amino acids and derivatives, organic acids, and benzene/substituted derivatives forming major metabolite classes [17]. This treatment upregulated tyrosine metabolism, downregulated starch and sucrose metabolism, and identified core metabolites associated with enhanced nutrient and Cd accumulation [17].

This metabolic pattern is biologically meaningful. Upregulation of amino-acid and phenolic pathways can stabilize membranes and provide precursors for antioxidant and chelating compounds, whereas redistribution of carbohydrate metabolism may prioritize defense and detoxification over unrestricted growth. In the same Cd system, glutathione (GSH) increased in leaves and roots and was associated with Cd localization in cell-wall fractions, supporting the idea that metabolic rewiring promotes both tolerance and phytoremediation [17]. Under salinity and drought, the same logic applies: proline, soluble sugars, amino acids, and organic acids buffer osmotic stress and protect enzymes and membranes, while phenolics and flavonoids contribute to ROS scavenging and stress signaling [18,19,25].

### 7.4. Proteomic and Enzyme-Level Adaptations in Host and Fungus

Proteomic and enzyme-level evidence adds a second layer of resolution, as stress tolerance depends not only on transcript abundance but also on protein stability, post-translational regulation, and enzyme activity. In the plant host, *Trichoderma* often increases antioxidant enzymes, including SOD, CAT, POD, APX, and GR, while reducing H_2_O_2_, MDA, and lipid peroxidation [19,25,69]. These enzymes form the biochemical execution layer of redox regulation, translating transcriptional and metabolic changes into cellular protection.

The fungal side of the interaction is equally important because an inoculant must remain viable before it can influence a crop. Heat-treated *T. harzianum* strains showed enhanced fungal post-recovery growth, 50–58% polymorphism, and HSP bands of approximately 120 and 131 kDa [68]. These findings concern fungal adaptation and should not be used as direct evidence of crop heat tolerance or field performance. Candidate strains identified through fungal stress screening require subsequent plant-level tests that measure colonization, physiological injury, reproductive performance, and yield under realistic temperature regimes. Additional fungal genetic evidence from secreted proteins, hydrophobins, and cell-wall-associated factors is discussed below with explicit separation among fungal mutants, purified-protein or peptide assays, heterologous plant expression, and direct inoculation.

### 7.5. Experimentally Tested Trichoderma Genes and Proteins: Functional Evidence and Experimental Boundaries

Expression-level associations are useful for screening, but the strongest functional evidence comes from direct genetic perturbation or purified protein or peptide assays. Swollenin (*swo1*) from *T. asperellum* is a representative example. Fungal overexpression accelerated early cucumber-root colonization, whereas silencing reduced colonization; overexpression of a construct lacking the 36-amino-acid carbohydrate-binding domain (CBD) segment did not reproduce the enhanced colonization phenotype. A synthetic 36-mer peptide derived from the CBD induced local defense responses and local protection in cucumber [75]. Importantly, the defense-active peptide was CBD-derived, not the N-terminal secretion signal peptide. Thus, swollenin links plant-surface interactions, fungal establishment, and defense elicitation, but fungal transformation and peptide assays should not be conflated with outcomes of routine inoculation.

Other genes and proteins illustrate distinct evidence categories. Deletion of *TasHyd1* in *T. asperellum* impaired spore attachment and root colonization, with genetic restoration recovering the phenotype [38]. In *T. virens*, *SM1* deletion reduced and *SM1* overexpression enhanced induced systemic protection in maize, demonstrating a direct fungal-gene contribution to defense [76]. For *ThKEL1*, experiments combined fungal silencing with heterologous overexpression in *Arabidopsis* and rapeseed; the results linked *ThKEL1* with Brassicaceae root colonization and jasmonic-acid-associated systemic defense, while transgenic expression also produced salt- and osmotic-stress phenotypes [77]. Separately, heterologous expression of a *T. harzianum* aquaglyceroporin gene in tobacco improved water-use efficiency and drought tolerance [61]. These transgenic or heterologous-expression studies constitute mechanistic proof-of-concept and must be distinguished explicitly from experiments in which crops receive direct *Trichoderma* inoculation. The representative genes and proteins, experimental systems, principal plant-associated findings, and corresponding boundaries of interpretation are summarized in Table 4.

### 7.6. Amplicon Sequencing, Microbiome Associations, and Network Inference

*Trichoderma*-mediated stress alleviation extends beyond host molecular reprogramming to treatment-associated changes in the rhizosphere microbiome; however, such evidence must be interpreted within the analytical scope and limitations of the methods employed [5,27,49]. Guzmán-Guzmán et al. [49] broadly highlighted the multifunctional involvement of *Trichoderma* in plant health and microbiome interactions. At the primary-study level, Wang et al. [5] employed metagenomic and functional-gene analyses to investigate microbiome responses under aged microplastic stress. By contrast, Yao et al. [27] used 16S rRNA gene and internal transcribed spacer amplicon sequencing, together with co-occurrence network analysis, to characterize microbial communities in Cd-contaminated soil cultivated with *B. juncea*. The combined application of *T. harzianum* and polyaspartic acid increased Cd-removal efficiency from 21.71% to 38.27%. It coincided with shifts in microbial community composition, network topology, soil-enzyme activities, and the occurrence of highly connected or core-associated taxa [27]. These findings nevertheless remain associative. Amplicon sequencing characterizes taxonomic composition, whereas co-occurrence networks identify statistical relationships among sequence features; neither approach, on its own, establishes causal interactions, ecological functions, network repair, or keystone status. Accordingly, taxa identified through centrality or recurrent core-occurrence criteria should be described as candidate hub taxa or core-associated taxa unless their ecological functions have been validated experimentally.

In the context of emerging pollutants, Wang et al. [5] further demonstrated that treatment of aged polybutylene adipate terephthalate microplastic-stressed *Nicotiana benthamiana* with *T. harzianum* T4 was associated with recovery of microbial diversity, increased relative abundances of Bacteroidota and Myxococcota, decreased relative abundances of *tetA5* and multidrug-resistance-related genes, and enrichment of carbohydrate-active enzyme genes. These patterns provide evidence of treatment-associated restructuring of microbial communities and functional-gene profiles, but do not establish that any individual taxon directly caused plant recovery. Future investigations should therefore integrate amplicon sequencing to characterize community composition, shotgun metagenomics to assess functional potential, metatranscriptomics to identify actively expressed pathways, and targeted isolation or synthetic-community experiments to test causality. These microbiome-level analyses should be coupled with detailed plant phenotyping to establish their physiological and agronomic relevance.

### 7.7. Pan-Genome-Guided Strain Selection for Climate-Resilient Bioformulations

The next step is to use omics not only to explain responses but also to design better *Trichoderma* technologies. Pan-genomic analysis of 25 agriculturally and industrially relevant *Trichoderma* strains identified an open pan-genome and 4960 shared core genes, with accessory and unique genes enriched in adaptive functions, defense-related traits, and secondary metabolism [32]. This supports a move from empirical isolate screening toward predictive strain selection. To mitigate abiotic stress, candidate strains should be screened for gene modules associated with osmotic tolerance, heat tolerance, antioxidant capacity, root colonization, phosphate transport, secondary metabolism, hydrophobins, secreted proteins, and compatibility with beneficial bacteria. Comparative genomic evidence also indicates extensive lateral acquisition of plant cell wall-degrading enzyme genes in *Trichoderma*, reinforcing that functionally relevant gene repertoires can be evolutionarily dynamic across lineages [78].

**Figure 3:**
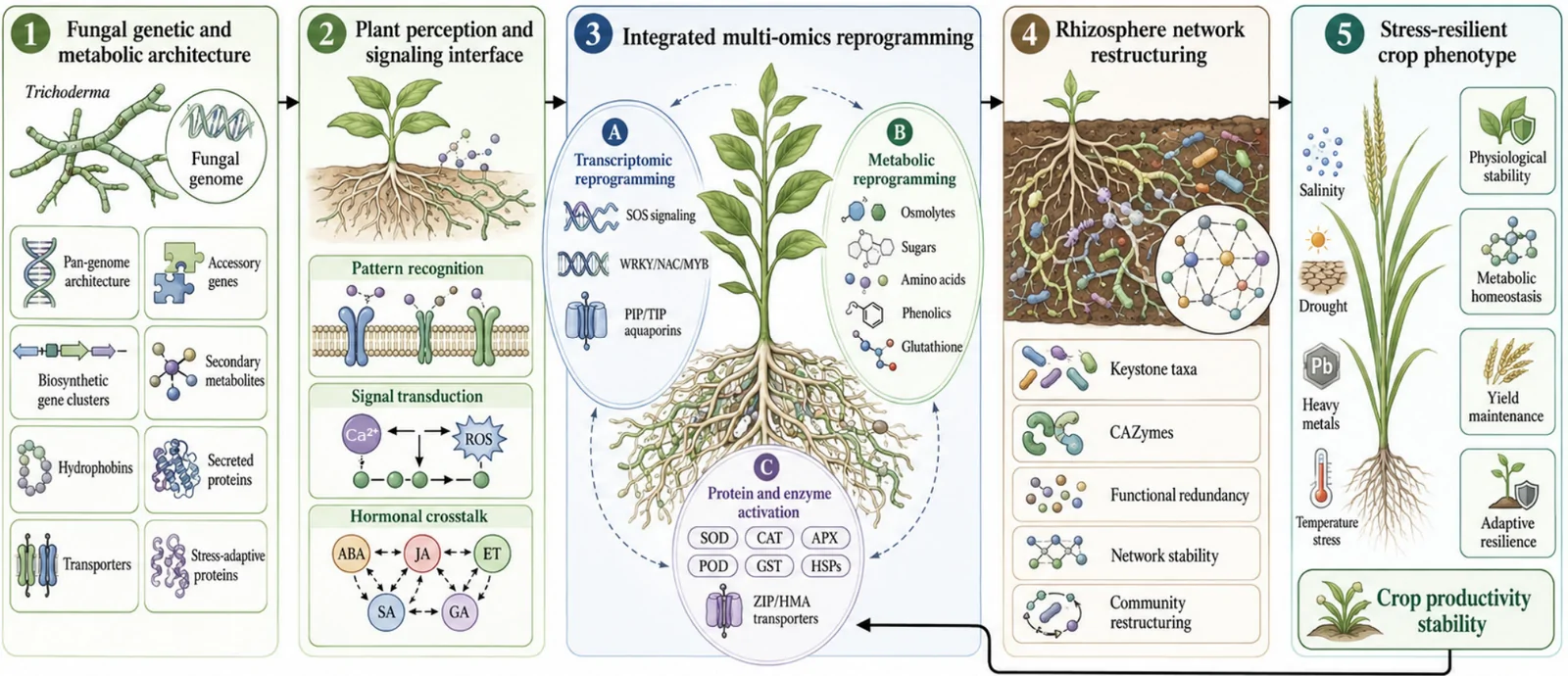
Molecular and multi-omics architecture of Trichoderma-mediated abiotic stress responses. The schematic presents five interconnected stages: (1) fungal genetic and metabolic architecture, including pan-genome organization, accessory genes, biosynthetic gene clusters, secondary metabolites, hydrophobins, secreted proteins, transporters, and stress-adaptive proteins; (2) plant perception and signaling through pattern recognition, calcium- and reactive-oxygen-species-mediated signal transduction, and hormonal crosstalk; (3) integrated multi-omics reprogramming involving transcriptomic, metabolic, and protein- or enzyme-level responses; (4) treatment-associated rhizosphere-network restructuring; and (5) development of a stress-resilient crop phenotype under salinity, drought, heavy-metal, and temperature stresses. The numbered and color-coded panels distinguish the major functional stages of the framework. Solid arrows indicate the proposed conceptual progression among these stages, dashed circular arrows indicate coordinated interactions among transcriptomic, metabolic, and protein- or enzyme-level responses, and bidirectional arrows indicate regulatory crosstalk or reciprocal interactions. Icons and molecular symbols represent the biological components or processes identified by their adjacent labels. Colors are used solely for visual organization and do not represent quantitative effects, treatment intensity, statistical significance, or universally established causal relationships. In the rhizosphere panel, candidate hub taxa, predicted functions, and inferred network descriptors represent hypotheses generated from sequencing and co-occurrence analyses rather than experimentally established keystone functions, causal network repair, or ecological stability. Crop-level temperature and yield outcomes require direct validation. Original author-created schematic; not adapted from published material. Details of AI-assisted preliminary layout and author-led finalization are provided in the Declaration of Generative AI and AI-Assisted Technologies.

A predictive systems-biology pipeline should integrate genome and pan-genome screening, crop-specific transcriptomics and metabolomics, protein or enzyme assays, and method-appropriate microbiome analysis. Amplicon sequencing can profile bacterial and fungal community composition; shotgun metagenomics can characterize functional potential; metatranscriptomics can identify active pathways; and co-occurrence networks can generate hypotheses about statistical associations. Figure 3 therefore presents microbiome topology as an inferential layer rather than evidence of causal network repair. These tools should be linked to direct measurements of colonization, crop physiology, and harvest outcomes before strains or formulations are advanced for field testing. This molecular perspective changes how *Trichoderma* should be evaluated. A strain that increases biomass in a pot experiment is not automatically suitable for field use. Candidate technologies should also demonstrate viable delivery, reproducible root colonization, crop-specific stress responses, biosafety, and agronomic benefit across relevant environments. Molecular and omics tools can prioritize candidates, but they do not replace multi-location field validation or justify claims of reliable field performance on their own.

## 8. Rhizosphere Engineering and Microbial Consortia

Abiotic stress reshapes the rhizosphere before it is fully expressed as a yield penalty. Drought limits water films and nutrient diffusion, salinity changes osmotic potential and ion exchange, heavy metals suppress sensitive microbial groups, and temperature extremes alter microbial metabolism. These soil-level changes can weaken the plant’s ability to recruit beneficial microorganisms and acquire resources. For this reason, *Trichoderma* should be interpreted not only as a plant-growth-promoting fungus but also as a rhizosphere engineer: a biological agent that can reorganize root-associated microbial interactions, nutrient cycling, soil enzyme activity, and stress-buffering functions. This ecological view is essential for explaining field variability. A strain that performs well in a pot experiment may fail in a field soil if it cannot persist, communicate with resident microbiota, or become embedded in the root-zone network.

### 8.1. Inter-Kingdom Communication at the Fungal-Bacterial Interface

The rhizosphere is organized through physical contact and chemical exchange. *Trichoderma* hyphae provide surfaces for bacterial attachment, biofilm development, and dispersal through soil pores, allowing motile bacteria to use fungal networks as biological corridors towards roots or nutrient-rich microsites [41]. This hyphosphere effect is particularly relevant in dry or structurally heterogeneous soils, where bacterial movement is otherwise restricted. At the same time, bacteria can influence fungal growth, sporulation, enzyme production, and metabolite expression, creating bidirectional regulation rather than a one-way fungal effect [41]. Chemical communication adds a second layer of control. *Trichoderma* releases volatile organic compounds, sesquiterpenes, peptaibols, enzymes, and diffusible metabolites that may stimulate, inhibit, or reprogramme bacterial partners [33,35]. For example, volatile compounds from *Trichoderma atroviride* have been reported to alter expression of biocontrol-related genes in *Pseudomonas fluorescens*, while bacterial metabolites can modulate fungal endochitinase activity and other traits linked to antagonism [41]. Such inter-kingdom signaling can strengthen plant protection, but it can also create antagonism if one partner suppresses the other. Therefore, fungal-bacterial interaction should be treated as a selectable ecological trait, not an automatic benefit of co-inoculation. Under abiotic stress, this crosstalk becomes more important because plants must simultaneously perform multiple functions: nutrient mobilization, osmotic buffering, oxidative stress control, pathogen suppression, and root system maintenance. A single isolate may provide only part of this functional portfolio. Compatible consortia can distribute functions among partners only when their metabolic and spatial interactions are cooperative. This principle separates rational consortium design from empirical mixing of beneficial isolates.

### 8.2. Synergistic Partners: Bacillus, Pseudomonas, Rhizobia, and Plant-Growth-Promoting Bacteria

Plant-growth-promoting bacteria broaden the functional range of *Trichoderma*-based technologies. *Bacillus* spp. contribute stress-tolerant endospores, lipopeptides, hydrolytic enzymes, volatile compounds, and biofilm-forming traits, traits that support persistence under dry or nutrient-limited conditions. *Pseudomonas* spp. are efficient rhizosphere colonizers that produce siderophores, antibiotics, indole-3-acetic acid (IAA), and phosphorus-solubilizing metabolites. Rhizobia, including *Rhizobium* and *Bradyrhizobium*, provide nitrogen-fixation capacity, while other plant-growth-promoting bacteria, such as *Azospirillum* and *Azotobacter*, can support root development and nutrient cycling [79,80]. Evidence for synergy is strongest in plant growth promotion and disease management, but the same functional logic is relevant to abiotic stress. In a recent synthesis of *Trichoderma*-bacterial interactions, field and greenhouse studies showed that some consortia increased photosynthetic pigments, mineral content, antioxidant traits, and nutritional value while reducing ROS products [41]. A talc-based formulation combining *T. harzianum* CBF2 with *Pseudomonas aeruginosa* DRB1 reduced Fusarium-related disease symptoms by 58% and improved viability, illustrating how formulation and partner compatibility can determine biological performance [41]. Although this example is disease-oriented, the underlying principles—stable survival, complementary metabolism, and rhizosphere persistence—are directly relevant to abiotic stress inoculants. In legumes and oilseed crops, *Trichoderma*-rhizobia interactions are particularly attractive because nitrogen fixation, root growth, and stress tolerance are tightly linked. However, genotype-to-genotype compatibility can determine whether a consortium becomes synergistic or antagonistic [41]. This means that combinations involving *Trichoderma*, rhizobia, and other plant-growth-promoting bacteria should be screened under the target crop, soil, and stress conditions rather than assumed to be universally effective.

### 8.3. Biochar as a Soil Amendment, Microbial Habitat, and Candidate Carrier Material

Biochar can modify soil water-holding capacity, cation-exchange capacity, nutrient retention, microsite heterogeneity, pH, and metal mobility; however, the magnitude and direction of these effects depend strongly on feedstock type, pyrolysis conditions, particle size, surface chemistry, application rate, and soil properties [81,82]. Its porous structure may also provide protected microsites for microbial establishment. Nevertheless, the presence of microbial habitat does not, by itself, demonstrate that biochar functions as an effective microbial carrier. A genuine biochar-based *Trichoderma* formulation requires direct evidence that the inoculant was immobilized, adsorbed, encapsulated, or otherwise formulated on the material, together with measurements of propagule viability, storage stability, release dynamics, root-zone colonization, and carrier-specific effects.

Kipçak Bitik et al. [67] did not evaluate a biochar-immobilized fungal formulation. In their pepper experiment, biochar was incorporated independently as a soil amendment, whereas the *T. harzianum* suspension was applied separately. The study therefore supports an amendment–microbe co-application strategy rather than a carrier-mediated delivery mechanism. Under severe water deficit, the combined treatment was associated with increased antioxidant enzyme activities, lower H_2_O_2_ levels, greater proline and sucrose accumulation, and marked changes in phytohormone profiles. However, MDA concentrations were substantially higher than those in the water-stressed control, indicating that enhanced enzymatic ROS regulation did not correspond to reduced lipid peroxidation [67]. Similarly, Sofy et al. [69] investigated the integrated application of biochar and *T. harzianum* in salt-stressed spinach but did not provide formulation-specific evidence of fungal immobilization, protected delivery, or carrier-mediated persistence.

Future carrier studies should therefore include biochar-only, free-inoculum, and biochar-immobilized-inoculum treatments, together with measurements of shelf life, propagule viability, release kinetics, rhizosphere colonization, and plant responses across contrasting soils. Until such evidence is available, biochar should be described as a soil amendment, microbial habitat, or candidate carrier platform rather than as an experimentally validated *Trichoderma* carrier in these crop systems. The experimental distinction between independent co-application and genuine carrier formulation is consistent with the designs reported by Kipçak Bitik et al. [67] and Sofy et al. [69].

### 8.4. Microbial Co-Occurrence Networks, Candidate Hub Taxa, and Functional Hypotheses

Microbial co-occurrence networks summarize statistical associations and topological patterns derived from community-sequencing data. Network descriptors such as positive cohesion, modularity, natural connectivity, robustness, and node centrality can be valuable for identifying treatment-associated patterns and generating testable ecological hypotheses. However, these metrics do not, in isolation, demonstrate microbial cooperation, causal interactions, functional redundancy, ecological stability, network repair, or keystone function [83,84]. Accordingly, taxa exhibiting high centrality, recurrent occurrence, or strong connectivity should be described as candidate hub taxa, high-connectivity taxa, or core-associated taxa unless their ecological functions have been established experimentally.

In Cd-contaminated soil cultivated with *Brassica juncea*, Yao et al. [27] reported that the combined application of *T. harzianum* and polyaspartic acid increased Cd-removal efficiency and was associated with changes in microbial community composition, network topology, soil-enzyme activities, and the relative prominence of *Trichoderma*, *Pseudomonas*, and *Arenimonas*. These findings identify treatment-associated community and network patterns, but they do not establish that these taxa directly caused Cd removal, plant recovery, or greater ecological stability. Likewise, predicted functional enrichment and cross-network associations remain inferential unless they are confirmed through isolate manipulation, synthetic-community reconstruction, gene-expression analysis, functional assays, or direct measurements of ecological processes. The practical objective of consortium development should therefore not be framed as presumed “network repair.” Instead, it should focus on the reproducible assembly and delivery of compatible microorganisms whose persistence, functional contributions, interactions, and crop benefits have been demonstrated under clearly defined conditions. Yao et al. [27] used amplicon sequencing and co-occurrence analysis, which support association-based interpretation rather than causal ecological conclusions.

### 8.5. Risks, Context Dependence, and Design Principles

Although synthetic microbial consortia may combine complementary functions, they also introduce risks of strain incompatibility, unstable colonization, competitive exclusion, functional redundancy, and unintended effects on non-target microbial groups. Their performance can be strongly influenced by soil moisture, pH, organic-matter content, salinity, temperature, pesticide exposure, nutrient availability, indigenous microbiota, and stress intensity [29,30,85,86,87]. A consortium that performs effectively in sterile substrate or under controlled greenhouse conditions may therefore fail to establish or express the same functions in heterogeneous field soils.

Candidate consortia should progress through a staged validation framework that includes pairwise and community-level compatibility testing, functional-trait assays, formulation and storage evaluation, root-colonization measurements, greenhouse experiments, and multi-location field trials. Each omics method should be used according to its analytical capacity. Amplicon sequencing can characterize bacterial and fungal community composition; shotgun metagenomics can estimate functional-gene potential; metatranscriptomics can identify actively expressed pathways; and metabolomics can characterize chemical exchange among plants, fungi, and associated microorganisms. However, controlled isolate experiments, gene-function assays, and synthetic-community reconstruction are required to test causal interactions and functional contributions [79,83,88]. This sequential framework prevents statistical network correlations from being interpreted prematurely as experimentally validated ecological mechanisms.

**Figure 4:**
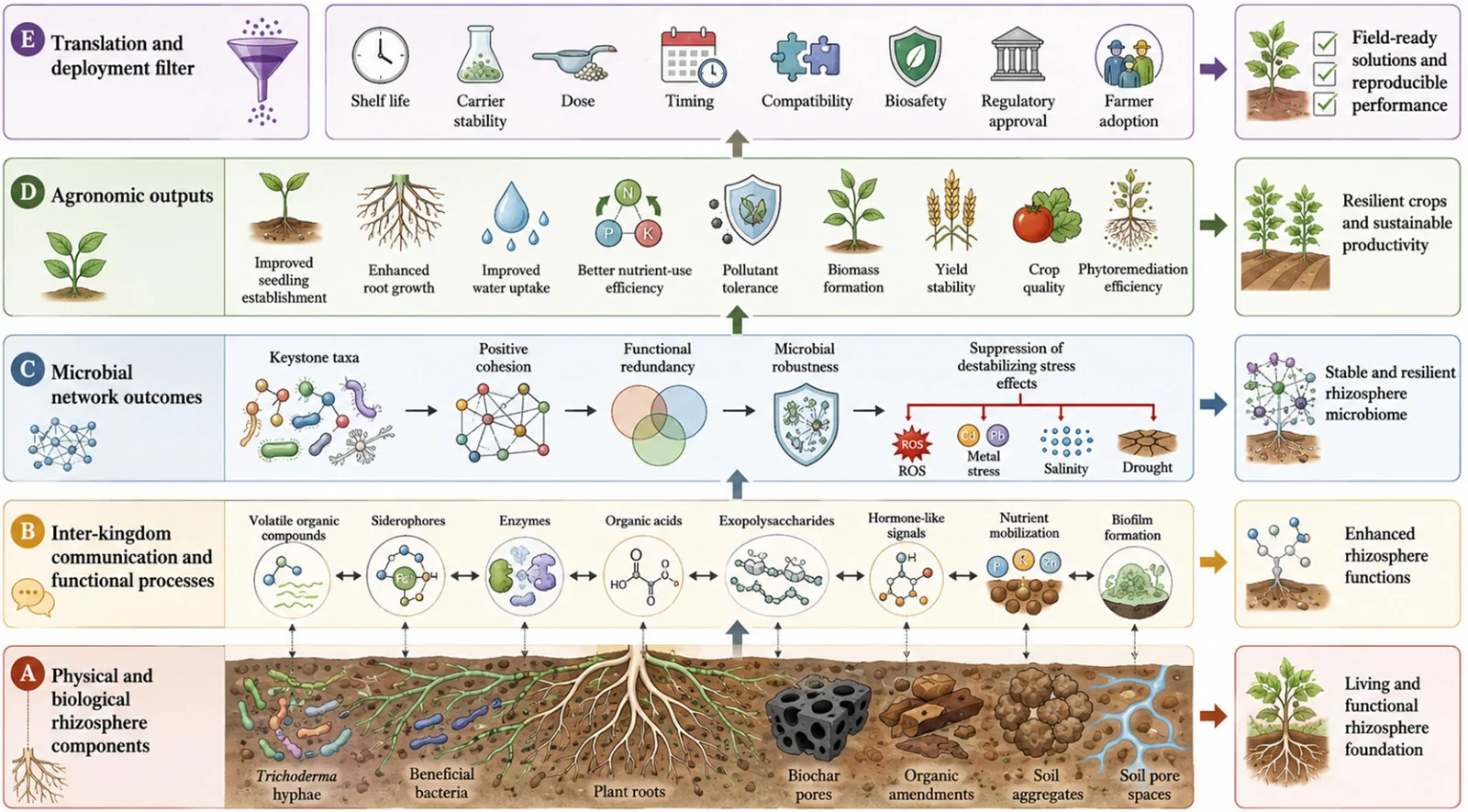
Rhizosphere engineering and field translation of *Trichoderma*-based crop-resilience technologies. The schematic distinguishes physical rhizosphere components and measured functional processes from inferred network descriptors and candidate agronomic outcomes. Candidate hub taxa, positive cohesion, predicted functional overlap, and inferred robustness are hypotheses generated from community and network analyses; they do not establish causality, keystone function, or ecological repair. Claims of field readiness and reproducible performance require direct multi-location validation of crop and yield outcomes. Original author-created schematic; not adapted from published material. Details of AI-assisted preliminary layout and author-led finalization are provided in the Declaration of Generative AI and AI-Assisted Technologies.

Figure 4 integrates these principles into a rhizosphere-engineering and field-translation framework. Physical and biochemical interactions among plant roots, *Trichoderma* hyphae, beneficial bacteria, biochar, organic amendments, and soil structure can influence microbial community composition and plant performance. In the figure, network metrics, candidate hub taxa, predicted functional overlap, and inferred robustness should be interpreted as analytical descriptors and hypothesis-generating outputs rather than as evidence of causal network stability or repair. Likewise, improvements in water uptake, nutrient acquisition, pollutant tolerance, biomass, crop quality, or yield must be verified directly within each crop–strain–soil–stress combination. Broader claims of agronomic reliability require reproducible evidence from multi-location and multi-season field trials.

## 9. Translating Mechanisms into Crop Productivity

The translational value of *Trichoderma*-mediated stress mitigation is determined by whether molecular, physiological, and rhizosphere-level responses are converted into agronomic gains. For farmers and production systems, the decisive outcomes are not only higher antioxidant enzyme activity or altered gene expression, but improved crop establishment, root growth, canopy formation, harvestable yield, product quality, nutrient-use efficiency, and stability under variable field conditions. The evidence summarized in Table 1, Table 2 and Table 3 and Figure 1, Figure 2, Figure 3 and Figure 4 supports a layered productivity model: formulation and delivery determine inoculum entry; root-rhizosphere establishment determines biological contact; physiological and omics-level reprogramming determine stress recovery; and rhizosphere engineering determines whether these effects persist long enough to influence yield. The productivity layer should therefore be evaluated through an evidence chain linking viable formulation, root establishment, physiological recovery, rhizosphere stability and harvestable output, while broader conceptual and translational gaps are addressed in the final research agenda.

### 9.1. Early Establishment, Seedling Vigor, and Root-System Architecture

Seedling establishment is the first agronomic checkpoint at which *Trichoderma* can influence crop performance. Abiotic stress can reduce germination, delay emergence, and restrict early root growth, leaving seedlings with limited capacity to explore soil water and nutrients. *Trichoderma* seed treatment, seed biopriming, root dipping, and rhizosphere inoculation can improve this phase by stimulating radicle protrusion, lateral root formation, root hair development, and early shoot growth [18,21,24,85]. These early effects are agronomically important because small differences in root growth during establishment can alter later canopy development, nutrient acquisition, and stress escape. Several lines of evidence link early *Trichoderma* responses with productivity-relevant traits. In common bean (*Phaseolus vulgaris* L.) exposed to combined salinity and *Sclerotinia sclerotiorum* stress, *T. harzianum* increased germination under combined stress to 96.0–97.0%, compared with 47.5–50.0% in untreated stressed plants, and markedly reduced damping-off [57]. These data show that early vigor can integrate abiotic stress tolerance with disease suppression. In seed- and root-associated systems, beneficial fungi can also improve seed performance under osmotic, salt, heat, and cold stress, partly through rapid effects on imbibition and by eliciting volatile compounds that stimulate seedling growth [21,44,85]. Such responses should be interpreted as productivity-enabling traits: they do not guarantee yield, but they increase the probability that the crop enters the vegetative phase with a functional root system.

### 9.2. Vegetative Growth, Biomass Accumulation, and Canopy Function

After establishment, crop productivity depends on sustained root activity, leaf expansion, photosynthetic capacity, and biomass partitioning. Drought, salinity, and metal toxicity reduce leaf area, chlorophyll content, stomatal conductance, and nutrient uptake, thereby limiting carbon assimilation [4,6,7,10]. *Trichoderma* can partially reverse these constraints by increasing root absorptive capacity, stabilizing ion balance, reducing oxidative injury, and improving nutrient acquisition [18,19,25,70]. In Indian mustard (*Brassica juncea* L.) grown in naturally saline soil, *T. harzianum* increased chlorophyll, proline, nutrient uptake, and growth, while reducing sodium (Na^+^) accumulation and the Na^+^/potassium (K^+^) ratio [25]. This response is productivity-relevant because it connects stress physiology with canopy function and nutrient status rather than showing only a biochemical marker. The same logic applies to contaminated and degraded soils, where biomass is both a productivity endpoint and a prerequisite for remediation. In cadmium (Cd)-stressed Indian mustard, combined *T. harzianum* and polyaspartic acid increased total biomass by 42.38%, root volume by 30.65%, and total Cd accumulation by 79.11% [17]. A companion study reported that soil Cd removal efficiency increased from 21.71% to 38.27% when *T. harzianum* and polyaspartic acid were combined [27]. These results are important because they show that *Trichoderma* can improve dual agronomic functions: maintaining plant growth under toxic conditions while increasing the biological removal of a contaminant. In such systems, productivity should be evaluated as stress-resilient biomass plus functional output, rather than yield alone.

### 9.3. Harvest Outcomes, Crop Quality, and Nutrient-Use Efficiency

Harvestable output is the strongest test of agronomic value, but evidence must be reported at the scale actually studied. In saline Indian mustard, compost-based *T. harzianum* application increased seed yield by approximately 23% relative to untreated controls and increased oil content by 19.0–23.4% under stress [25]. These results support yield and quality benefits in that defined crop, treatment, and environment; they do not establish broad yield stability across crops or locations. *Trichoderma* may also support nutrient acquisition and carbon assimilation [18,25,39]. Still, fertilizer-replacement or reliable yield-protection claims require field-scale trials that report input reduction, nutrient balance, harvest outcomes, and economics across seasons and sites.

### 9.4. The Pot-to-Field Performance Filter

A central reason for inconsistent microbial products is the performance gap between controlled experiments and field agriculture. Pot studies often use uniform substrates, controlled irrigation, defined stress intensity, high inoculum density, and reduced microbial competition. Field soils are spatially heterogeneous and contain fluctuating moisture and salinity gradients, variable temperatures, pesticide residues, fertilizer history, and indigenous microbial communities. These factors can alter propagule survival, root colonization, and persistence [29,30,85,86]. Thus, field translation should be viewed as a performance filter rather than a simple scale-up step. Three forms of stability determine whether a *Trichoderma* treatment can move from greenhouse efficacy to agronomic reliability. Biological stability refers to the survival of a product during production, storage, and application. Ecological stability refers to colonization and persistence in the root zone despite competition from resident microbiota. Agronomic stability refers to the persistence of plant benefits through yield-determining stages. Because formulation is part of the mechanism, it also serves as a translational filter: if the inoculant loses viability, fails to reach the rhizosphere, or acts only during early vegetative growth, its effects may not translate into yield. The most convincing productivity studies are therefore those that combine colonization measurements, physiological markers, and harvest outcomes under field-relevant stress gradients.

### 9.5. Commercial Deployment: Formulation, Dose, Timing, and Farmer Adoption

Commercial value begins when a biological effect can be converted into a stable, affordable, and easy-to-use product. Key variables include propagule type, viable count, contamination control, carrier material, moisture content, shelf life, dispersibility, and compatibility with farm inputs [29,30,85,86,89]. Seed coatings, dry granules, and liquid formulations have distinct operational niches. Biochar co-application may be useful in degraded or water-limited soils, but the cited co-application studies [67,69] should not be described as carrier formulations. A biochar-based carrier requires direct evidence of immobilization, storage viability, release, and root colonization relative to biochar-only and free-inoculum controls. Dose, timing, cost, application simplicity, and compatibility with existing practices must be evaluated before a candidate product can be described as field-ready.

### 9.6. Biosafety, Persistence, and Responsible Productivity Claims

Biosafety is an integral component of translation because strain identity, ecological persistence, non-target effects and quality control determine whether biological efficacy can be responsibly converted into field use. *Trichoderma* spp. are widely used as plant-beneficial fungi, but field deployment still requires strain-level identification, quality control, contaminant exclusion, ecological monitoring, and assessment of non-target effects. Introduced inoculants can alter resident microbial communities, and these shifts may be beneficial, neutral, or undesirable depending on soil context and management [87]. Environmental persistence is similarly double-edged: persistence is needed for reliable function, but uncontrolled persistence or unintended spread should be evaluated, particularly for multicomponent inoculants and bioformulations used across diverse agroecosystems [90]. Responsible productivity claims should therefore be based on an evidence chain rather than isolated growth responses. At minimum, the chain should connect viable formulation, root or rhizosphere establishment, physiological recovery, and harvestable output. Stronger evidence would also include quality traits, nutrient-use efficiency, microbiome response, environmental persistence, and farmer-level economics. This evidence chain keeps the productivity discussion focused on field translation, while the remaining agenda requires standardization, multi-location validation, combined-stress testing, omics-guided strain selection and regulatory alignment.

## 10. Challenges, Research Gaps, and Future Prospects

Current evidence supports *Trichoderma* spp. as a promising biological platform for crop resilience under abiotic stress, but its field reliability remains constrained by strain specificity, crop genotype, formulation quality, soil context and stress complexity. Yet, the field has reached a point where further demonstrations of growth promotion are less important than understanding why responses are reproducible in some systems and inconsistent in others. The main challenge is translational. It involves converting strain-level potential into reliable agronomic performance through root-rhizosphere establishment, molecular stress reprogramming, and microbiome effects. A forward agenda must focus on precision strain selection, field validation, formulation standardization, realistic combined-stress testing, ecological safety, and farmer-level feasibility.

### 10.1. From Broad Claims to Strain-Crop-Stress Specificity

A central limitation is the tendency to describe benefits at the genus or species level. *T. harzianum*, *T. asperellum*, *T. virens*, and related species are frequently reported to improve stress tolerance. However, their performance is strongly strain-dependent [21,28,31,32]. Closely related isolates can differ in root colonization, secondary-metabolite production, nutrient mobilization, hydrophobin expression, stress tolerance, and compatibility with resident microbiota [31,32,37,38]. Thus, statements such as “*Trichoderma* improves drought tolerance” or “*T. harzianum* mitigates salinity” should be supported by strain identity, crop genotype, stress intensity, application route, and soil context. Crop specificity is equally important. A strain that improves salt tolerance in Indian mustard (*Brassica juncea* L.) may not produce the same magnitude or mechanism of response in rice (*Oryza sativa* L.), wheat (*Triticum aestivum* L.), tomato (*Solanum lycopersicum* L.), or citrus rootstocks. Host plants differ in root exudation, nutrient demand, hormone sensitivity, and microbiome recruitment [18,21,25,56]. Future studies should therefore be structured as strain-by-crop-by-stress matrices rather than single-strain, single-host assays. Such designs would help identify whether a strain is broadly stress-adaptive, crop-specific, or suitable only for a defined stress niche.

### 10.2. Long-Term and Multi-Location Field Validation

Pot and greenhouse experiments are essential for mechanistic discovery, but they cannot fully predict field performance. Controlled systems often use simplified soils, uniform stress exposure, regular irrigation, and high inoculum density. Field soils are more heterogeneous. They contain fluctuating moisture, pH, temperature, organic matter, nutrient availability, pesticide residues, and indigenous microbial competitors [29,30,85,86,87]. These variables affect fungal survival, root colonization, microbiome integration, and plant response. Thus, the transition from controlled efficacy to field reliability remains a major gap in *Trichoderma* research. Future trials should be multi-location, multi-season, and soil-type aware. Field studies should measure yield and quality, as well as inoculum viability, colonization persistence, root-zone localization, soil enzyme activity, microbiome shifts, and actual stress intensity throughout the crop cycle [29,30,85,86,87,88,89,90]. Long-term experiments are particularly needed for saline soils, heavy-metal-contaminated systems, perennial crops, and repeated inoculant application. Without such data, it is difficult to distinguish a transient biostimulant effect from a durable crop-resilience technology.

### 10.3. Standardization of Formulation, Dose, and Timing

Formulation determines whether *Trichoderma* remains viable, reaches the root zone, and colonizes the target crop. Current studies vary in propagule type, inoculum density, storage period, application route, and crop stage [29,30]. Seed coating, biopriming, root dipping, soil drenching, compost incorporation, free liquid inoculum, biochar co-application, and experimentally immobilized carrier systems should be distinguished rather than grouped. Future work should report viable propagule density, shelf life, carrier composition, moisture tolerance, compatibility with inputs, and timing relative to stress. Carrier-only, free-inoculum, and immobilized-inoculum controls are essential because amendments can independently affect water retention, nutrient supply, and microbial activity. A carrier claim is justified only when the carrier function itself has been tested.

### 10.4. Realistic Combined-Stress Systems and Recovery Phases

Most experiments examine a single stress. In contrast, farming systems commonly expose crops to simultaneous or sequential stresses. Drought can coincide with heat. Salinity can interact with nutrient imbalance. Heavy-metal toxicity can overlap with poor soil structure, pH stress, or pathogen pressure [3,11]. Combined biotic-abiotic stress is also agronomically important. For example, salinity and *Sclerotinia sclerotiorum* together reduce common bean (*Phaseolus vulgaris* L.) performance [57]. Single-stress studies may overestimate inoculant reliability. *Trichoderma* studies should increasingly use factorial designs to test whether responses are additive, synergistic, antagonistic, or stress-specific. The field also needs more attention to recovery. Crop productivity is shaped not only by the ability to withstand stress, but also by the speed and completeness of recovery after stress release. Measurements after re-watering, salt leaching, temperature normalization, or post-contamination growth could indicate whether *Trichoderma* improves resilience trajectories rather than simply reducing damage at a single sampling time.

### 10.5. Predictive Omics, Synthetic Consortia, Biochar Co-Application, and Carrier Development

Omics approaches can support the rational development of *Trichoderma*-based technologies, but their outputs must be interpreted in light of each method’s resolution and limitations. Pan-genomics can identify candidate fungal genes and strain-specific functional repertoires; transcriptomics and proteomics can characterize stress-responsive changes in the host and fungus; metabolomics can reveal compounds associated with signaling and stress adaptation; amplicon sequencing can describe microbial community composition; shotgun metagenomics can estimate community functional potential; and co-occurrence-network analysis can generate hypotheses regarding statistical relationships among microbial taxa [5,27,49,79,91,92,93]. These approaches are valuable for candidate prioritization and mechanistic hypothesis generation, but none independently demonstrate causal ecological function, agronomic effectiveness, or field reliability.

Omics-guided synthetic consortia should therefore undergo systematic evaluation of strain compatibility, functional complementarity, formulation stability, root colonization, persistence, and reproducibility across crop, soil, and stress conditions. Similar evidentiary distinctions are required for biochar-based applications. Biochar used independently as a soil amendment, biochar co-applied with a freely applied microbial suspension, and a true biochar-immobilized fungal formulation represent experimentally distinct delivery systems. Kipçak Bitik et al. [67] evaluated biochar as a separately incorporated soil amendment and *T. harzianum* as an independently applied suspension in water-stressed pepper, whereas Sofy et al. [69] examined their integrated application in salt-stressed spinach. Consequently, both studies support amendment–microbe co-application but do not demonstrate immobilization of *Trichoderma* on biochar or a carrier-mediated protection mechanism. Future carrier studies should verify inoculant immobilization, propagule viability during storage, release dynamics, root-zone colonization, carrier-only effects, plant responses, and performance across multiple soils before attributing observed benefits to carrier-mediated delivery.

### 10.6. Biosafety, Ecological Persistence, Regulation, and Farmer Economics

Although *Trichoderma* is widely used and generally regarded as beneficial, biosafety should be evaluated at the strain level. Introduced strains may differ in persistence, metabolite production, host range, and effects on non-target microorganisms [21,31,33]. Repeated applications, protected cultivation, contaminated soils, and multi-strain consortia require particular attention because they may reshape resident microbiomes. Monitoring should include persistence of the introduced strain, non-target microbial shifts, potential suppression of beneficial fungi or bacteria, and compatibility with local agroecosystems. Regulatory approval and farmer adoption are equally important. A microbial product may be biologically effective. However, it can be commercially weak if it has poor shelf life, inconsistent performance, complex application requirements, or high cost. Product development should include cost per hectare, yield return, reduction in fertilizer or pesticide use, labor requirements, storage conditions, and compatibility with irrigation, seed treatment, or nursery systems [86,89,94,95,96,97]. Farmer-level economics should not be treated as a final step in commercialization. Instead, it should guide formulation and field-trial design from the beginning.

### 10.7. Perspective: Towards Precision Microbial Resilience Technologies

The next phase of *Trichoderma* research should shift from documenting growth promotion to developing precision microbial technologies. Candidate strains or consortia should be matched to a defined crop, soil, stress profile, formulation, and management system. Products proposed for saline vegetables, drought-prone cereals, metal-contaminated soils, or heat-stressed nurseries require stress-specific plant phenotyping, colonization assays, formulation validation, biosafety evaluation, and multi-location field trials. Fungal survival under heat exposure is a selection criterion, not evidence of crop heat protection. Only after reproducible plant and harvest outcomes are demonstrated should a technology be described as field-ready or reliable.

## 11. Conclusions

*Trichoderma* spp. are promising, context-dependent regulators of crop responses to abiotic stress. The strongest crop-level evidence currently concerns salinity, drought, and heavy-metal systems and links root development, photosynthesis, antioxidant regulation, osmotic adjustment, nutrient acquisition, ion homeostasis, and stress-responsive gene expression. Microbiome studies report treatment-associated changes in community composition, functional-gene profiles, and co-occurrence-network descriptors, but these analyses do not, by themselves, prove causal network repair, keystone function, or ecological stability. Evidence for broad heat and cold protection remains limited, and fungal survival after temperature exposure must be distinguished from crop protection. Likewise, biochar co-application studies should not be interpreted as carrier formulations without direct formulation evidence. Progress toward reliable use therefore requires strain- and crop-specific testing, standardized formulation and carrier validation, mechanistic experiments that test causality, multi-location field trials, biosafety assessment, and farmer-level economic evaluation.

## Figures and Tables

**Table 1:** Functional traits of *Trichoderma* linking genomic capacity, formulation, and abiotic stress mitigation. Summarizes the genomic, metabolic, secreted, nutritional, and formulation-related traits through which *Trichoderma* connects strain-level potential with plant stress resilience and field performance.

Functional Layer	Evidence Base	Contribution to Abiotic Stress Resilience	Ref.
Pan-genome architecture	An open pan-genome across 25 agriculturally and industrially relevant *Trichoderma* strains, including 4960 shared core genes and variable accessory and strain-specific gene complements.	Provides the genetic basis for strain-specific adaptation, rhizosphere fitness, secondary metabolism, root interaction and selection of stress-resilient inoculants.	[32]
Biosynthetic gene clusters and secondary metabolism	Diverse biosynthetic gene clusters (BGCs), including non-ribosomal peptide synthetase (NRPS), polyketide synthase (PKS), terpene, peptaibol and hybrid metabolite systems.	Generate chemical mediators involved in microbial competition, plant signaling, defense priming, root modulation and stress-response activation.	[31,32,33,34]
Volatile organic compounds and 6-PP	*Trichoderma* volatile organic compounds (VOCs) show broad chemical diversity; 6-pentyl-2H-pyran-2-one (6-PP) from *T. atroviride* regulates *Arabidopsis thaliana* root morphogenesis through auxin and ethylene signaling.	Promote early root-system remodeling and stress preparedness, even before extensive physical colonization is established.	[35,36]
Secreted enzymes and small secreted proteins	Genome-era analyses show enrichment of cell-wall-degrading enzymes and small secreted proteins involved in fungal interaction, mycoparasitism and host communication.	Support nutrient turnover, rhizosphere remodeling, microbial competition, elicitation of plant responses and functional persistence in the root zone.	[31,33]
Hydrophobins and surface-interface proteins	HFB7 is induced by biotic and abiotic stresses in Harzianum and Virens clades, whereas TasHyd1 from *T. asperellum* contributes to plant root colonization.	Link fungal attachment, surface adhesion, interface formation and environmental persistence with stable plant association under stress.	[37,38]
Nutrient mobilization capacity	*T. harzianum* Rifai 1295-22 solubilizes phosphates and micronutrients, indicating direct involvement in mineral mobilization.	Improves nutrient availability and uptake under drought, salinity and metal-stressed soils, where nutrient diffusion and root acquisition are restricted.	[39]
Bioformulation and carrier systems	Shelf life, propagule viability, carrier quality and ecosystem adaptation determine the transition from laboratory efficacy to field performance.	Controls whether genetic and metabolic traits remain functional after storage, transport, soil application and exposure to environmental stress.	[29,30]
Microbial consortia	*Trichoderma* can be combined with beneficial bacteria such as *Bacillus* and *Pseudomonas*, although compatibility and formulation stability are decisive.	Expands functional capacity through complementary nutrient cycling, biofilm formation, pathogen suppression and stress buffering, but requires careful strain compatibility testing.	[28,40,41]

Abbreviations: BGC, biosynthetic gene cluster; NRPS, non-ribosomal peptide synthetase; PKS, polyketide synthase; VOC, volatile organic compound; 6-PP, 6-pentyl-2H-pyran-2-one.

**Table 2:** Stress-specific evidence for *Trichoderma*-mediated abiotic stress mitigation.

Stress Domain	Crop or System	Stress Injury and Context	*Trichoderma* Intervention	Measured Response or Output	Mechanistic Interpretation	Refs.
Salinity	Indian mustard (*Brassica juncea* L.)	Natural saline soil reduced chlorophyll, nutrient uptake and yield while increasing ROS and MDA.	*T. harzianum* compost or suspension.	Compost increased seed yield by ~23%; oil content increased by 19–23.4%; Na^+^/K^+^ ratio in Tori-7 declined from 2.14 to ~0.92 under the strongest compost treatment.	Improved ionic balance, antioxidant activity, chlorophyll retention and nutrient assimilation.	[25]
Salinity	Indian mustard (*Brassica juncea* L.)	NaCl stress reduced growth and disturbed mineral balance.	*T. harzianum* inoculation.	Improved uptake of essential elements and antioxidant defense under NaCl stress.	Antioxidant activation and nutrient stabilization under salt stress.	[58]
Salinity	*Arabidopsis thaliana* sos1 mutant	150 mM NaCl; the sos1 background is hypersensitive to Na^+^ stress.	*T. harzianum* root association.	Fresh weight, chlorophyll fluorescence, photosynthetic pigments and ROS-scavenging transcripts increased; proline, alanine, sucrose and glucose accumulated; Na^+^ accumulation was restricted.	Compensation of salt sensitivity through osmolytes, redox regulation and Na^+^ restriction.	[55]
Salinity	Citrus rootstock	Salt stress inhibited seedling growth and induced nutrient imbalance.	*T. harzianum* inoculation.	Plant height, stem diameter, leaf number, biomass, photosynthetic rate, stomatal conductance and chlorophyll increased; Na decreased; SOS, PIP and TIP genes were up-regulated.	Enhanced Na^+^ efflux, water transport, photosynthesis and nutrient uptake.	[56]
Combined salinity–biotic stress	Common bean (*Phaseolus vulgaris* L.)	Salinity plus *Sclerotinia sclerotiorum* reduced germination to 47.5–50.0% and increased damping-off to 50.0–52.5%.	*T. harzianum* and *T. koningii*.	*T. harzianum* increased germination to 96.0–97.0% and reduced damping-off to 10.8–14.5%; oxidative and membrane-damage markers declined.	Integrated control of ion toxicity, oxidative stress and pathogen-associated damage.	[57]
Drought	Rice (*Oryza sativa* L.)	Water deficit impairs germination, seedling growth and molecular stress programming.	*T. harzianum* biopriming.	Drought-challenged bioprimed rice showed altered molecular programming associated with improved drought response.	Early microbial priming can precondition later water-deficit responses.	[60]
Drought	Tobacco (*Nicotiana tabacum* L.)	Drought limits water-use efficiency and turgor maintenance.	Aquaglyceroporin gene from *T. harzianum.*	Overexpression improved water-use efficiency and drought tolerance.	Fungal water-channel traits can contribute to plant water-use physiology.	[61]
Drought	Maize (*Zea mays* L.)	Drought reduces crop development and nutrient acquisition.	*T. asperellum* T34 seed application.	T34-treated seeds maintained fungal populations and protected maize against drought stress.	Seed-applied fungal establishment supports drought tolerance through sustained root-zone activity.	[62]
Drought	Sugarcane (*Saccharum officinarum* L.)	Drought reduces photosynthesis, stomatal conductance and water-use efficiency.	*T. asperellum* inoculation.	Improved crop nutrition, chlorophyll/carotenoid content, photosynthetic rate, stomatal conductance, water-use efficiency, SOD/POD activity, proline and sugar partitioning.	Coupled photosynthetic recovery with antioxidant and osmotic adjustment.	[63]
Drought + co-application	Pepper (*Capsicum annuum* L.)	Water deficit reduced nutrient uptake and increased H_2_O_2_ and MDA.	Separate biochar soil amendment plus independently applied *T. harzianum* suspension; not a carrier.	At 50% irrigation: CAT, POD and SOD > 40%; H_2_O_2_ ~25% lower; MDA ~49.4 vs. 16.8 mg g^−1^ fresh weight.	Discordant redox response. Pot co-application evidence does not demonstrate carrier-mediated protection or field performance.	[67]
Heavy metal/phytoremediation	Indian mustard (*Brassica juncea* L.) under Cd stress	Cd damages roots, chloroplasts and membranes while limiting biomass and nutrient acquisition.	*T. harzianum* + polyaspartic acid.	Photosynthetic parameters increased; root volume +30.65%; biomass +42.38%; total Cd accumulation +79.11%; leaf Cd +71.12%.	Growth restoration and nutrient-Cd co-transport increased tolerance and removal capacity.	[17]
Heavy metal/detoxification	Indian mustard (*Brassica juncea* L.) under Cd stress	Cd induces oxidative damage and requires compartmentalization.	*T. harzianum* + polyaspartic acid.	Cd localized mainly in cell wall and vacuolar/soluble fractions; GSH increased 23.62% in leaves and 32.12% in roots; 3525 root metabolites were detected.	Antioxidant protection, GSH-linked detoxification and metabolic reprogramming support Cd tolerance.	[17]
Heavy metal/rhizosphere associations	Indian mustard (*Brassica juncea* L.) under Cd stress	Cd remediation assessed with soil chemistry, 16S rRNA/ITS amplicons and co-occurrence analysis.	*T. harzianum* + polyaspartic acid.	Cd removal increased 21.71–38.27%; community composition/topology and core or high-connectivity taxa shifted; ZIP and TC.HME transporters increased.	Statistical associations only; no demonstrated causal network repair, keystone function or ecological stability.	[27]
Temperature/fungal adaptation	*T. harzianum*; no crop-level experiment	Heat can reduce inoculant viability and biocontrol performance.	Heat-treated, recovered *T. harzianum* strains.	Enhanced fungal post-stress growth; 50–58% polymorphism; HSP bands at ~120 and 131 kDa.	Fungal adaptation only; crop heat protection, yield benefit and field performance remain untested.	[68]
Emerging pollutants	Tobacco relative (*Nicotiana benthamiana*)	Aged PBAT microplastics inhibited growth, increased ROS/MDA and disrupted metabolic homeostasis.	*T. harzianum* T4.	ROS/MDA decreased; SOD/POD increased; biomass improved; *Bacteroidota* and *Myxococcota* increased; tetA5/MDR genes decreased; CAZymes increased.	Plant stress-response activation plus microbiome and functional-gene recovery.	[5]
Salinity + co-application	Spinach (*Spinacia oleracea* L.)	Salinity reduces water uptake, mineral balance and redox stability.	Separate biochar soil amendment plus independently applied *T. harzianum*.	Reported amelioration of salt stress through improved physiological and biochemical status.	Co-application was associated with improved plant status; the study does not demonstrate biochar-mediated fungal carrying or persistence.	[69]

Abbreviations: ABA, abscisic acid; APX, ascorbate peroxidase; CAZyme, carbohydrate-active enzyme; CAT, catalase; Cd, cadmium; GA, gibberellic acid; GSH, glutathione; H_2_O_2_, hydrogen peroxide; HSP, heat-shock protein; IAA, indole-3-acetic acid; MDA, malondialdehyde; PBAT, polybutylene adipate terephthalate; PIP, plasma membrane intrinsic protein; POD, peroxidase; ROS, reactive oxygen species; SOD, superoxide dismutase; SOS, salt overly sensitive; TIP, tonoplast intrinsic protein.

**Table 3:** Physiological and biochemical markers reprogrammed by *Trichoderma* under abiotic stress. The table summarizes measurable crop markers that connect stress injury with downstream growth recovery and can be used to compare results across crops, stress types and formulations.

Mechanistic Axis	Primary Stress Injury	*Trichoderma*-Regulated Markers	Representative Quantitative Evidence and Interpretation	Refs.
Photosynthetic recovery	Chlorophyll loss; reduced gas exchange, fluorescence and carbon assimilation	Chlorophyll, carotenoids, stomatal conductance, net photosynthesis, photosystem protection	Indian mustard: chlorophyll and oil content increased under salinity and compost delivery increased seed yield by 23%. Cd-stressed *Brassica* showed chlorophyll +37.10–91.98%, carotenoids +34.78–72.37% and biomass +42.38%, indicating that photosynthetic protection is directly linked with growth recovery.	[17,25,70]
Redox buffering	ROS burst, H_2_O_2_ accumulation and oxidative injury	SOD, CAT, POD, APX, GR, GSH, AsA, phenolics, flavonoids	Pepper under 50% irrigation: biochar-*T. harzianum* co-application increased CAT, POD and SOD by >40% and reduced H_2_O_2_ by ~25%, but MDA increased substantially (~49.4 vs. 16.8 mg g^−1^ fresh weight). Cd-stressed *Brassica* showed leaf CAT +158.89%, SOD +50.82% and POD +6.71%.	[17,25,67,73]
Osmotic adjustment	Cell dehydration, reduced turgor and protein instability	Proline, sucrose, glucose, alanine, soluble sugars, amino acids, soluble proteins	*Arabidopsis* sos1 mutants accumulated proline, alanine, sucrose and glucose after *T. harzianum* inoculation. Pepper co-application increased proline and sucrose, but the independently applied biochar and fungal suspension should not be described as a carrier formulation.	[55,67]
Nutrient acquisition	Restricted nutrient diffusion and uptake under drought, salinity or metal stress	N, P, S, K, Ca, Mg, Zn, Cu; phosphate and micronutrient mobilization	Indian mustard showed improved N, P, S, Ca, Mg and K uptake under salinity; citrus seedlings showed higher N, P, Ca, Mg, Zn and Cu and lower Na under salt stress.	[25,39,56]
Ion homeostasis	Na^+^ and Cl^−^ toxicity; low K^+^ retention; disrupted Na^+^/K^+^ ratio	Na^+^ exclusion, K^+^ retention, Na^+^/K^+^ balance, Ca^2+^ signaling	Indian mustard showed lower Na uptake and a lower Na^+^/K^+^ ratio, while *Trichoderma*-treated *Arabidopsis* sos1 plants restricted Na^+^ accumulation and improved salt-stress performance.	[25,55,56]
Metal detoxification and phytoremediation	Cd-, Pb-, As- or Cr-induced ROS, nutrient disorder and organelle injury	GSH, phytochelatin precursors, cell-wall binding, vacuolar sequestration, nutrient-metal co-transport	Cd-stressed *Brassica* showed biomass +42.38%, leaf Cd +71.12% and preferential Cd localization in cell wall and vacuolar/soluble fractions, supporting tolerance and phytoremediation.	[17]
Membrane stability	Lipid peroxidation, electrolyte leakage, MDA accumulation	MDA, H_2_O_2_, electrolyte leakage, osmolytes and antioxidant pools; markers must be interpreted jointly	Several studies reported lower MDA/H_2_O_2_, but Kipçak Bitik et al. [67] reported lower H_2_O_2_ together with substantially higher MDA. It therefore supports partial redox regulation, not generalized reduction in lipid peroxidation.	[17,25,26,67]
Hormonal balance	Stress-induced growth arrest or maladaptive defense activation	IAA, GA, ABA, SA, JA, ET	Pepper co-application produced large IAA, GA, ABA, SA and JA shifts in a controlled pot study. These data do not establish a biochar-based fungal carrier or field-level hormonal recovery.	[67,72]
Genotype and strain dependence	Variable responses across crop genotypes and fungal isolates	Colonization rate, biomarker magnitude, stress-response specificity	Tomato water-deficit studies show isolate- and genotype-dependent drought relief; barley salt studies indicate genotype-specific biochemical pathways after *T. harzianum* T-22 inoculation.	[71,74]

Abbreviations: ABA, abscisic acid; APX, ascorbate peroxidase; AsA, ascorbate; BTH, biochar plus *Trichoderma harzianum*; CAT, catalase; ET, ethylene; GA, gibberellic acid; GR, glutathione reductase; GSH, reduced glutathione; H_2_O_2_, hydrogen peroxide; IAA, indole-3-acetic acid; JA, jasmonic acid; MDA, malondialdehyde; POD, peroxidase; ROS, reactive oxygen species; SA, salicylic acid; SOD, superoxide dismutase.

**Table 4:** Representative *Trichoderma* genes and proteins tested by direct genetic manipulation, purified-protein or peptide assays, or heterologous expression, with explicit limits on inference to inoculation-based crop responses.

Gene or Protein	Experimental Test and System	Principal Plant-Associated Finding	Evidence Category and Boundary
Swollenin (*swo1*)	*T. asperellum* overexpression and silencing; CBD-deletion construct; synthetic 36-mer CBD peptide in cucumber	Early root colonization depended on swollenin; the CBD-derived peptide elicited local defense and protection.	Fungal genetics plus peptide assay; the active peptide was not the secretion signal [75].
*TasHyd1*	*T. asperellum* deletion, overexpression and restoration strains in cucumber	Required for efficient spore attachment and root colonization.	Direct fungal mutant and complementation evidence [38].
*SM1*	*T. virens* deletion and overexpression during maize interaction	*SM1* loss reduced, and overexpression enhanced, induced systemic protection.	Direct fungal-gene manipulation with inoculation [76].
*ThKEL1*	*T. harzianum* silenced transformants plus heterologous expression in *Arabidopsis* and rapeseed	Linked to Brassicaceae root colonization, JA-associated systemic defense and transgenic stress phenotypes.	Mixed fungal-mutant and transgenic-plant evidence; not equivalent to routine inoculation [77].
Aquaglyceroporin	Heterologous expression of a *T. harzianum* gene in *Nicotiana tabacum*	Improved water-use efficiency and drought tolerance.	Transgenic plant proof-of-concept, not direct fungal inoculation [61].

## Data Availability

No new data were created or analyzed in this study. Data sharing is not applicable to this review article.

## Abbreviations

6-PP, 6-pentyl-2H-pyran-2-one; 16S rRNA, 16S ribosomal RNA; ABA, abscisic acid; APX, ascorbate peroxidase; As, arsenic; AsA, ascorbate; BGC, biosynthetic gene cluster; BTH, biochar plus *Trichoderma harzianum*; CAZyme, carbohydrate-active enzyme; CAT, catalase; CBD, carbohydrate-binding domain; Cd, cadmium; CO_2_, carbon dioxide; DNA, deoxyribonucleic acid; ET, ethylene; GA, gibberellic acid; GR, glutathione reductase; GSH, reduced glutathione; H_2_O_2_, hydrogen peroxide; HMA, heavy-metal ATPase; HSP, heat-shock protein; IAA, indole-3-acetic acid; ISR, induced systemic resistance; ITS, internal transcribed spacer; JA, jasmonic acid; MAPK, mitogen-activated protein kinase; MDA, malondialdehyde; MDR, multidrug resistance; NRPS, non-ribosomal peptide synthetase; PASP, polyaspartic acid; PBAT, polybutylene adipate terephthalate; PIP, plasma membrane intrinsic protein; PKS, polyketide synthase; POD, peroxidase; PR, pathogenesis-related; ROS, reactive oxygen species; SA, salicylic acid; SAR, systemic acquired resistance; SOD, superoxide dismutase; SOS, salt overly sensitive; TIP, tonoplast intrinsic protein; VOC, volatile organic compound; ZIP, zinc-regulated transporter/iron-regulated transporter-like protein.

## References

[1] Kopecká R., Kameniarová M., Černý M., Brzobohatý B., Novák J. (2023). Abiotic Stress in Crop Production. Int. J. Mol. Sci..

[2] Jeyasri R., Muthuramalingam P., Satish L., Pandian S.K., Chen J.T., Ahmar S., Wang X., Mora-Poblete F., Ramesh M. (2021). An Overview of Abiotic Stress in Cereal Crops: Negative Impacts, Regulation, Biotechnology and Integrated Omics. Plants.

[3] Zandalinas S.I., Fritschi F.B., Mittler R. (2021). Global Warming, Climate Change, and Environmental Pollution: Recipe for a Multifactorial Stress Combination Disaster. Trends Plant Sci..

[4] Munns R., Tester M. (2008). Mechanisms of Salinity Tolerance. Annu. Rev. Plant Biol..

[5] Wang F., Sun X., Wang K., Long B., Li F., Xie D. (2025). Physiological and Multi-Omics Insights into *Trichoderma harzianum* Alleviating Aged Microplastic Stress in *Nicotiana benthamiana*. Int. J. Mol. Sci..

[6] Farooq M., Wahid A., Kobayashi N., Fujita D., Basra S.M.A. (2009). Plant drought stress: Effects, mechanisms and management. Agron. Sustain. Dev..

[7] Gill S.S., Tuteja N. (2010). Reactive oxygen species and antioxidant machinery in abiotic stress tolerance in crop plants. Plant Physiol. Biochem..

[8] Hasanuzzaman M., Nahar K., Fujita M. (2013). Plant Response to Salt Stress and Role of Exogenous Protectants to Mitigate Salt-Induced Damages. Ecophysiology and Responses of Plants Under Salt Stress.

[9] Wahid A., Gelani S., Ashraf M., Foolad M. (2007). Heat tolerance in plants: An overview. Environ. Exp. Bot..

[10] Haider F.U., Liqun C., Coulter J.A., Cheema S.A., Wu J., Zhang R., Wenjun M., Farooq M. (2021). Cadmium toxicity in plants: Impacts and remediation strategies. Ecotoxicol. Environ. Saf..

[11] Sánchez-Bermúdez M., Del Pozo J.C., Pernas M. (2022). Effects of Combined Abiotic Stresses Related to Climate Change on Root Growth in Crops. Front. Plant Sci..

[12] Tardieu F. (2012). Any trait or trait-related allele can confer drought tolerance: Just design the right drought scenario. J. Exp. Bot..

[13] Mickelbart M.V., Hasegawa P.M., Bailey-Serres J. (2015). Genetic mechanisms of abiotic stress tolerance that translate to crop yield stability. Nat. Rev. Genet..

[14] Seleiman M.F., Al-Suhaibani N., Ali N., Akmal M., Alotaibi M., Refay Y., Dindaroglu T., Abdul-Wajid H.H., Battaglia M.L. (2021). Drought Stress Impacts on Plants and Different Approaches to Alleviate Its Adverse Effects. Plants.

[15] Kopittke P.M., Menzies N.W., Wang P., McKenna B.A., Lombi E. (2019). Soil and the intensification of agriculture for global food security. Environ. Int..

[16] Godoy F., Olivos-Hernández K., Stange C., Handford M. (2021). Abiotic Stress in Crop Species: Improving Tolerance by Applying Plant Metabolites. Plants.

[17] Yao S., Zhou B., Yu P., Bi Y., Ren P., Duan M., Chen X. (2025). Synergistic regulation of Cd stress tolerance in *Brassica juncea*: Metabolic reprogramming and nutrient-Cd co-transport under *Trichoderma harzianum* and polyaspartic acid. Environ. Chem. Ecotoxicol..

[18] Contreras-Cornejo H.A., Schmoll M., Esquivel-Ayala B.A., González-Esquivel C.E., Rocha-Ramírez V., Larsen J. (2024). Mechanisms for plant growth promotion activated by *Trichoderma* in natural and managed terrestrial ecosystems. Microbiol. Res..

[19] Boorboori M.R., Zhang H. (2023). The Mechanisms of *Trichoderma* Species to Reduce Drought and Salinity Stress in Plants. Phyton-Int. J. Exp. Bot..

[20] Geng Y., Chen S., Lv P., Li Y., Li J., Jiang F., Wu Z., Shen Q., Zhou R. (2025). Positive Role of *Trichoderma harzianum* in Increasing Plant Tolerance to Abiotic Stresses: A Review. Antioxidants.

[21] Woo S.L., Hermosa R., Lorito M., Monte E. (2023). *Trichoderma*: A multipurpose, plant-beneficial microorganism for eco-sustainable agriculture. Nat. Rev. Microbiol..

[22] Yakhin O.I., Lubyanov A.A., Yakhin I.A., Brown P.H. (2017). Biostimulants in Plant Science: A Global Perspective. Front. Plant Sci..

[23] Harman G.E. (2011). Multifunctional fungal plant symbionts: New tools to enhance plant growth and productivity. New Phytol..

[24] Harman G.E., Howell C.R., Viterbo A., Chet I., Lorito M. (2004). *Trichoderma* species—Opportunistic, avirulent plant symbionts. Nat. Rev. Microbiol..

[25] Saha K.C., Uddin M.K., Shaha P.K., Hossain Chowdhury M.A., Hassan L., Saha B.K. (2025). Application of *Trichoderma harzianum* enhances salt tolerance and yield of Indian mustard through increasing antioxidant enzyme activity. Heliyon.

[26] Mastouri F., Björkman T., Harman G.E. (2012). *Trichoderma harzianum* enhances antioxidant defense of tomato seedlings and resistance to water deficit. Mol. Plant-Microbe Interact..

[27] Yao S., Zhou B., Duan M., Yu P., Bi Y., Ren P., Chen X. (2025). Synergistic effects of *Trichoderma harzianum* and polyaspartic acid enhance cadmium phytoremediation via core microbiota and network stability in *Brassica juncea*. Environ. Chem. Ecotoxicol..

[28] Kredics L., Büchner R., Balázs D., Allaga H., Kedves O., Racić G., Varga A., Nagy V.D., Vágvölgyi C., Sipos G. (2024). Recent advances in the use of *Trichoderma*-containing multicomponent microbial inoculants for pathogen control and plant growth promotion. World J. Microbiol. Biotechnol..

[29] Bejarano A., Puopolo G. (2020). Bioformulation of Microbial Biocontrol Agents for a Sustainable Agriculture. How Research Can Stimulate the Development of Commercial Biological Control Against Plant Diseases.

[30] Teixidó N., Segarra G., Casals C., Usall J., Torres R. (2020). Formulations to Improve Biocontrol Products Shelf-Life and/or Ecosystem Adaptation. How Research Can Stimulate the Development of Commercial Biological Control Against Plant Diseases.

[31] Mukherjee P.K., Horwitz B.A., Herrera-Estrella A., Schmoll M., Kenerley C.M. (2013). *Trichoderma* research in the genome era. Annu. Rev. Phytopathol..

[32] Mondal A., Parvez S.S., Bera D., Alam M., Banik A. (2026). *Trichoderma* in multitrophic plant-microbe interactions: A pan-genome guided roadmap for resilient physiology and sustainable bio-economy. Plant Physiol. Biochem..

[33] Khan R.A., Najeeb S., Hussain S., Xie B., Li Y. (2020). Bioactive Secondary Metabolites from *Trichoderma* spp. Against Phytopathogenic Fungi. Microorganisms.

[34] Vinale F., Sivasithamparam K., Ghisalberti E.L., Woo S.L., Nigro M., Marra R., Lombardi N., Pascale A., Ruocco M., Lanzuise S. (2014). *Trichoderma* secondary metabolites active on plants and fungal pathogens. Open Mycol. J..

[35] Guo Y., Jud W., Ghirardo A., Antritter F., Benz J.P., Schnitzler J.P., Rosenkranz M. (2020). Sniffing fungi-phenotyping of volatile chemical diversity in *Trichoderma* species. New Phytol..

[36] Garnica-Vergara A., Barrera-Ortiz S., Muñoz-Parra E., Raya-González J., Méndez-Bravo A., Macías-Rodríguez L., Ruiz-Herrera L.F., López-Bucio J. (2016). The volatile 6-pentyl-2H-pyran-2-one from *Trichoderma atroviride* regulates *Arabidopsis thaliana* root morphogenesis via auxin signaling and ETHYLENE INSENSITIVE 2 functioning. New Phytol..

[37] Przylucka A., Akcapinar G.B., Chenthamara K., Cai F., Grujic M., Karpenko J., Livoi M., Shen Q., Kubicek C.P., Druzhinina I.S. (2017). HFB7-A novel orphan hydrophobin of the Harzianum and Virens clades of *Trichoderma*, is involved in response to biotic and abiotic stresses. Fungal Genet. Biol..

[38] Viterbo A., Chet I. (2006). TasHyd1, a new hydrophobin gene from the biocontrol agent *Trichoderma asperellum*, is involved in plant root colonization. Mol. Plant Pathol..

[39] Altomare C., Norvell W.A., Bjorkman T., Harman G.E. (1999). Solubilization of phosphates and micronutrients by the plant-growth-promoting and biocontrol fungus *Trichoderma harzianum* Rifai 1295-22. Appl. Environ. Microbiol..

[40] Poveda J., Eugui D. (2022). Combined use of *Trichoderma* and beneficial bacteria (mainly *Bacillus* and *Pseudomonas*): Development of microbial synergistic bio-inoculants in sustainable agriculture. Biol. Control.

[41] Mohd Din A.R.J., Mohamad Azam Z., Othman N.Z., Yong J.W. (2025). *Trichoderma*-bacterial network: A balance inter-kingdom interaction for agricultural relevance. Microbe.

[42] He C., Liu C., Liu H., Wang W., Hou J., Li X. (2022). Dual inoculation of dark septate endophytes and *Trichoderma viride* drives plant performance and rhizosphere microbiome adaptations of *Astragalus mongholicus* to drought. Environ. Microbiol..

[43] Yedidia I., Benhamou N., Chet I. (1999). Induction of defense responses in cucumber plants (*Cucumis sativus* L.) BY Biocontrol Agent *Trichoderma harzianum*. Appl. Environ. Microbiol..

[44] Hung R., Lee S., Bennett J.W. (2013). *Arabidopsis thaliana* as a model system for testing the effect of *Trichoderma* volatile organic compounds. Fungal Ecol..

[45] Contreras-Cornejo H.A., Macías-Rodríguez L., Cortés-Penagos C., López-Bucio J. (2009). *Trichoderma virens*, a Plant Beneficial Fungus, Enhances Biomass Production and Promotes Lateral Root Growth through an Auxin-Dependent Mechanism in *Arabidopsis*. Plant Physiol..

[46] Gravel V., Antoun H., Tweddell R.J. (2007). Growth stimulation and fruit yield improvement of greenhouse tomato plants by inoculation with *Pseudomonas putida* or *Trichoderma atroviride*: Possible role of indole acetic acid (IAA). Soil Biol. Biochem..

[47] Viterbo A., Landau U., Kim S., Chernin L., Chet I. (2010). Characterization of ACC deaminase from the biocontrol and plant growth-promoting agent *Trichoderma asperellum* T203. FEMS Microbiol. Lett..

[48] Velmourougane K., Prasanna R., Singh S., Chawla G., Kumar A., Saxena A.K. (2017). Modulating rhizosphere colonisation, plant growth, soil nutrient availability and plant defense enzyme activity through *Trichoderma viride*-*Azotobacter chroococcum* biofilm inoculation in chickpea. Plant Soil.

[49] Guzmán-Guzmán P., Etesami H., Santoyo G. (2025). *Trichoderma*: A multifunctional agent in plant health and microbiome interactions. BMC Microbiol..

[50] Berendsen R.L., Pieterse C.M.J., Bakker P.A.H.M. (2012). The rhizosphere microbiome and plant health. Trends Plant Sci..

[51] Cai F., Chen W., Wei Z., Pang G., Li R., Ran W., Shen Q. (2015). Colonization of *Trichoderma harzianum* strain SQR-T037 on tomato roots and its relationship to plant growth, nutrient availability and soil microflora. Plant Soil.

[52] Hermosa R., Viterbo A., Chet I., Monte E. (2012). Plant-beneficial effects of *Trichoderma* and of its genes. Microbiology.

[53] Brotman Y., Landau U., Cuadros-Inostroza Á., Tohge T., Fernie A.R., Chet I., Viterbo A., Willmitzer L. (2013). *Trichoderma*-plant root colonization: Escaping early plant defense responses and activation of the antioxidant machinery for saline stress tolerance. PLoS Pathog..

[54] Shoresh M., Harman G.E., Mastouri F. (2010). Induced systemic resistance and plant responses to fungal biocontrol agents. Annu. Rev. Phytopathol..

[55] Gandhi A., Reichelt M., Goyal D., Vadassery J., Oelmüller R. (2025). *Trichoderma harzianum* Protects the *Arabidopsis* Salt Overly Sensitive 1 Mutant Against Salt Stress. J. Plant Growth Regul..

[56] Zhao M., Wang P., Liu X., Jin L. (2026). The Effects of *Trichoderma harzianum* Inoculation on the Growth, Nutrient Absorption, and Expressions of Stress-Responsive Genes of Citrus Under Salt Stress. Horticulturae.

[57] Abdelrhim A.S., Hemeda N.F., Mwaheb M.A., Omar M.O., Dawood M.F. (2024). The role of *Trichoderma koningii* and *Trichoderma harzianum* in mitigating the combined stresses motivated by *Sclerotinia sclerotiorum* and salinity in common bean (*Phaseolus vulgaris*). Plant Stress.

[58] Ahmad P., Hashem A., Abd-Allah E.F., Alqarawi A.A., John R., Egamberdieva D., Gucel S. (2015). Role of *Trichoderma harzianum* in mitigating NaCl stress in Indian mustard (*Brassica juncea* L.) through the antioxidative defense system. Front. Plant Sci..

[59] Metwally R.A., Soliman S.A. (2023). Alleviation of the adverse effects of NaCl stress on tomato seedlings (*Solanum lycopersicum* L.) by *Trichoderma viride* through the antioxidative defense system. Bot. Stud..

[60] Bashyal B.M., Parmar P., Zaidi N.W., Aggarwal R. (2021). Molecular Programming of Drought-Challenged *Trichoderma harzianum*-Bioprimed Rice (*Oryza sativa* L.). Front. Microbiol..

[61] Vieira P.M., Santos M.P., Andrade C.M., Souza-Neto O.A., Ulhoa C.J., Aragão F.J.L. (2017). Overexpression of an aquaglyceroporin gene from *Trichoderma harzianum* improves water-use efficiency and drought tolerance in *Nicotiana tabacum*. Plant Physiol. Biochem..

[62] Estévez-Geffriaud V., Vicente R., Vergara-Díaz O., Reinaldo J.J.N., Trillas M.I. (2020). Application of *Trichoderma asperellum* T34 on maize (*Zea mays*) seeds protects against drought stress. Planta.

[63] Scudeletti D., Crusciol C.A., Bossolani J.W., Moretti L.G., Momesso L., Servaz Tubaña B., De Castro S.G., De Oliveira E.F., Hungria M. (2021). *Trichoderma asperellum* Inoculation as a Tool for Attenuating Drought Stress in Sugarcane. Front. Plant Sci..

[64] Csótó A., Tóth G., Riczu P., Zabiák A., Tarjányi V., Fekete E., Karaffa L., Sándor E. (2024). Foliar Spraying with Endophytic *Trichoderma* Biostimulant Increases Drought Resilience of Maize and Sunflower. Agriculture.

[65] Mona S.A., Hashem A., Abd-Allah E.F., Alqarawi A.A., Soliman D.W.K., Wirth S., Egamberdieva D. (2017). Increased resistance of drought by *Trichoderma harzianum* fungal treatment correlates with increased secondary metabolites and proline content. J. Integr. Agric..

[66] Racić G., Vukelić I., Prokić L., Ćurčić N., Zorić M., Jovanović L., Panković D. (2018). The influence of *Trichoderma brevicompactum* treatment and drought on physiological parameters, abscisic acid content and signalling pathway marker gene expression in leaves and roots of tomato. Ann. Appl. Biol..

[67] Kipçak Bitik S., Ete Aydemir Ö., Kocaman A., Turan M., Özkutlu F. (2026). Physiological and biochemical ameliorative effects of biochar, *Trichoderma harzianum*, and combined applications in *Capsicum annuum* L. under water stress. Plant Growth Regul..

[68] Ahmed H.A., Kasem L.M., Attaby H.S., Khalil N.M., El Amir D.A., Diab M.R., El-Baghdady M.M., Radwan K.H., Ibrahim A.E. (2025). Molecular and genetic adaptations of heat-treated *Trichoderma harzianum* for enhanced stress resilience and biocontrol efficiency. Ann. Microbiol..

[69] Sofy M., Mohamed H., Dawood M., Abu-Elsaoud A., Soliman M. (2022). Integrated usage of *Trichoderma harzianum* and biochar to ameliorate salt stress on spinach plants. Arch. Agron. Soil Sci..

[70] Harman G.E., Doni F., Khadka R.B., Uphoff N. (2021). Endophytic strains of *Trichoderma* increase plants’ photosynthetic capability. J. Appl. Microbiol..

[71] Rawal R., Scheerens J.C., Fenstemaker S.M., Francis D.M., Miller S.A., Benitez M.S. (2022). Novel *Trichoderma* Isolates Alleviate Water Deficit Stress in Susceptible Tomato Genotypes. Front. Plant Sci..

[72] Martínez-Medina A., Del Mar Alguacil M., Pascual J.A., Van Wees S.C. (2014). Phytohormone Profiles Induced by *Trichoderma* Isolates Correspond with Their Biocontrol and Plant Growth-Promoting Activity on Melon Plants. J. Chem. Ecol..

[73] Zhang S., Xu B., Gan Y. (2019). Seed Treatment with *Trichoderma longibrachiatum* T6 Promotes Wheat Seedling Growth under NaCl Stress Through Activating the Enzymatic and Nonenzymatic Antioxidant Defense Systems. Int. J. Mol. Sci..

[74] Gupta S.V., Smith P.M., Natera S.H., Roessner U. (2022). Biochemical Changes in Two Barley Genotypes Inoculated With a Beneficial Fungus *Trichoderma harzianum* Rifai T-22 Grown in Saline Soil. Front. Plant Sci..

[75] Brotman Y., Briff E., Viterbo A., Chet I. (2008). Role of Swollenin, an Expansin-Like Protein from *Trichoderma*, in Plant Root Colonization. Plant Physiol..

[76] Djonović S., Vargas W.A., Kolomiets M.V., Horndeski M., Wiest A., Kenerley C.M. (2007). A Proteinaceous Elicitor Sm1 from the Beneficial Fungus *Trichoderma virens* Is Required for Induced Systemic Resistance in Maize. Plant Physiol..

[77] Poveda J., Hermosa R., Monte E., Nicolás C. (2019). The *Trichoderma harzianum* Kelch Protein *ThKEL1* Plays a Key Role in Root Colonization and the Induction of Systemic Defense in Brassicaceae Plants. Front. Plant Sci..

[78] Druzhinina I.S., Chenthamara K., Zhang J., Atanasova L., Yang D., Miao Y., Rahimi M.J., Grujic M., Cai F., Pourmehdi S. (2018). Massive lateral transfer of genes encoding plant cell wall-degrading enzymes to the mycoparasitic fungus *Trichoderma* from its plant-associated hosts. PLoS Genet..

[79] Trivedi P., Leach J.E., Tringe S.G., Sa T., Singh B.K. (2020). Plant-microbiome interactions: From community assembly to plant health. Nat. Rev. Microbiol..

[80] Glick B.R. (2012). Plant Growth-Promoting Bacteria: Mechanisms and Applications. Scientifica.

[81] Lehmann J., Rillig M.C., Thies J., Masiello C.A., Hockaday W.C., Crowley D. (2011). Biochar effects on soil biota—A review. Soil Biol. Biochem..

[82] Sohi S.P., Krull E., Lopez-Capel E., Bol R. (2010). A review of biochar and its use and function in soil. Adv. Agron..

[83] Banerjee S., Schlaeppi K., van der Heijden M.G.A. (2018). Keystone taxa as drivers of microbiome structure and functioning. Nat. Rev. Microbiol..

[84] Herren C.M., McMahon K.D. (2017). Cohesion: A method for quantifying the connectivity of microbial communities. ISME J..

[85] O’Callaghan M. (2016). Microbial inoculation of seed for improved crop performance: Issues and opportunities. Appl. Microbiol. Biotechnol..

[86] Bashan Y., de-Bashan L.E., Prabhu S.R., Hernandez J.-P. (2014). Advances in plant growth-promoting bacterial inoculant technology: Formulations and practical perspectives (1998–2013). Plant Soil.

[87] Trabelsi D., Mhamdi R. (2013). Microbial inoculants and their impact on soil microbial communities: A review. BioMed Res. Int..

[88] Toju H., Peay K.G., Yamamichi M., Narisawa K., Hiruma K., Naito K., Fukuda S., Ushio M., Nakaoka S., Onoda Y. (2018). Core microbiomes for sustainable agroecosystems. Nat. Plants.

[89] Backer R., Rokem J.S., Ilangumaran G., Lamont J., Praslickova D., Ricci E., Subramanian S., Smith D.L. (2018). Plant Growth-Promoting Rhizobacteria: Context, Mechanisms of Action, and Roadmap to Commercialization of Biostimulants for Sustainable Agriculture. Front. Plant Sci..

[90] Sundh I., Goettel M.S. (2013). Regulating biocontrol agents: A historical perspective and a critical examination comparing microbial and macrobial agents. BioControl.

[91] Compant S., Samad A., Faist H., Sessitsch A. (2019). A review on the plant microbiome: Ecology, functions, and emerging trends in microbial application. J. Adv. Res..

[92] Finkel O.M., Castrillo G., Herrera Paredes S., Salas González I., Dangl J.L. (2017). Understanding and exploiting plant beneficial microbes. Curr. Opin. Plant Biol..

[93] Busby P.E., Soman C., Wagner M.R., Friesen M.L., Kremer J., Bennett A., Morsy M., Eisen J.A., Leach J.E., Dangl J.L. (2017). Research priorities for harnessing plant microbiomes in sustainable agriculture. PLoS Biol..

[94] Kumar S., Sindhu S.S., Kumar R. (2022). Biofertilizers: An ecofriendly technology for nutrient recycling and environmental sustainability. Curr. Res. Microb. Sci..

[95] Du Jardin P. (2015). Plant biostimulants: Definition, concept, main categories and regulation. Sci. Hortic..

[96] Malusa F., Vassilev N. (2014). A contribution to set a legal framework for biofertilisers. Appl. Microbiol. Biotechnol..

[97] Mitter E.K., Tosi M., Obregón D., Dunfield K.E., Germida J.J. (2021). Rethinking Crop Nutrition in Times of Modern Microbiology: Innovative Biofertilizer Technologies. Front. Sustain. Food Syst..

